# Aligned Ion Conduction Pathway of Polyrotaxane-Based Electrolyte with Dispersed Hydrophobic Chains for Solid-State Lithium–Oxygen Batteries

**DOI:** 10.1007/s40820-024-01535-w

**Published:** 2024-10-01

**Authors:** Bitgaram Kim, Myeong-Chang Sung, Gwang-Hee Lee, Byoungjoon Hwang, Sojung Seo, Ji-Hun Seo, Dong-Wan Kim

**Affiliations:** 1 Department of Materials Science and Engineering, Korea University, 145 Anam-Ro, Seongbuk-Gu, Seoul, 02841 Republic of Korea; 2 School of Civil, Environmental and Architectural Engineering, Korea University, 145 Anam-Ro, Seongbuk-Gu, Seoul, 02841 Republic of Korea; 3 Materials Science and Chemical Engineering Center, Institute for Advanced Engineering (IAE), Yongin, 17180 Republic of Korea

**Keywords:** Solid polymer electrolyte, Lithium-oxygen batteries, Polyrotaxane ion conductivity, Hydrophobic chain

## Abstract

A critical challenge hindering the practical application of lithium–oxygen batteries (LOBs) is the inevitable problems associated with liquid electrolytes, such as evaporation and safety problems. Our study addresses these problems by proposing a modified polyrotaxane (mPR)-based solid polymer electrolyte (SPE) design that simultaneously mitigates solvent-related problems and improves conductivity. mPR-SPE exhibits high ion conductivity (2.8 × 10^−3^ S cm^−1^ at 25 °C) through aligned ion conduction pathways and provides electrode protection ability through hydrophobic chain dispersion. Integrating this mPR-SPE into solid-state LOBs resulted in stable potentials over 300 cycles. In situ Raman spectroscopy reveals the presence of an LiO_2_ intermediate alongside Li_2_O_2_ during oxygen reactions. Ex situ X-ray diffraction confirm the ability of the SPE to hinder the permeation of oxygen and moisture, as demonstrated by the air permeability tests. The present study suggests that maintaining a low residual solvent while achieving high ionic conductivity is crucial for restricting the sub-reactions of solid-state LOBs.

**Supplementary Information:**

The online version contains supplementary material available at 10.1007/s40820-024-01535-w.

## Highlights

- Strategic materials design of polyrotaxane-based electrolytes was suggested by aligning the ion conduction pathways and dispersing hydrophobic chains for solid-state Li–O_2_ batteries.
- Owing to intentional design, solid-state Li–O_2_ battery resulted in stable potential over 300 cycles at 25 °C.

**Supplementary Information:**

The online version contains supplementary material available at 10.1007/s40820-024-01535-w.

## Graphical abstract

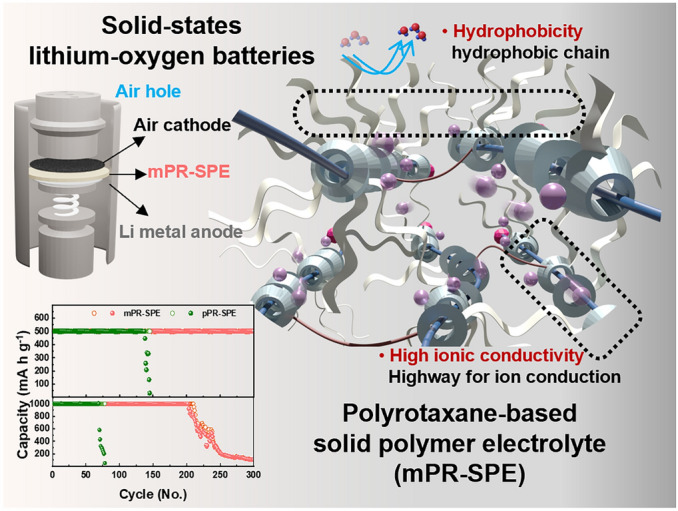

## Introduction

Rechargeable lithium-oxygen batteries (LOBs), which offer a high theoretical energy density of 3500 Wh kg^−1^, have emerged as a promising next-generation energy technology capable of satisfying the requirements of electric vehicles and energy storage systems [1]. LOB systems comprise a lithium anode, porous cathode, and an inorganic electrolyte-infiltrated separator that facilitates oxygen diffusion [2]. Primary reactions such as oxygen evolution/reduction occur primarily in organic liquid electrolytes (LEs). Presently, LEs contain carbonates, ethers, and esters capable of solvating Li salts such as LiAsF_6_, LiPF_6_, tetraethylene glycol dimethyl ether (TEGDME), and dimethylacetamide (DMAc) [3–5]. However, the use of LEs presents significant challenges, including dendrite growth, flammability, and low electrochemical stability. Additionally, conventional LEs are prone to nucleophilic attacks from lithium peroxide with no way to prevent the crossover of H_2_O and O_2_ in the anode direction, thereby affecting battery performance [6, 7]. These factors are pivotal challenges in the commercialization of LOBs.

Generally, two overarching methods are utilized to enhance the stability of LOBs: either by creating an artificial protective layer on the surface of lithium metal through the inclusion of additives in the liquid electrolyte or by substituting liquid electrolytes with a solid-state electrolyte. Huang et al. developed a protective film on lithium metal using boric acid as an additive in the liquid electrolyte [8]. Lee et al. introduced CoI_2_ into a liquid electrolyte, which mediated oxidation/reduction by iodine and simultaneously forms a stable cobalt protective layer for the Li anode [9]. In contrast to direct interventions that target lithium metal anodes in nonaqueous LOBs, solid-state electrolytes such as solid polymers and inorganic electrolytes offer distinct advantages. They can effectively mitigate lithium dendrite growth and O_2_ crossover, while concurrently addressing safety concerns related to flammable liquid electrolytes. Solid inorganic electrolytes (SIEs), a subset of solid-state electrolytes, exhibit high ion transport efficiency as single-ion cationic conductors [10]. However, SIEs face challenges due to their fragility and implementation complexity. To address these problems, the ductility and flexibility of SIEs are enhanced by applying a lithium-ion conductive polymer coating onto inorganic particles.

Solid polymer electrolytes (SPEs) have been investigated as alternatives, offering advantages such as enhanced safety, cost-effectiveness, and ease of processing. However, SPEs typically demonstrate lower ionic conductivity, compared to that of their counterparts, thus limiting practical application. The low ionic conductivity of SPEs is primarily attributed to the high degree of crystallization of the polymer chains, which impedes the segmental motion of the polymer matrix [11, 12]. Therefore, scholars have explored low-crystalline SPEs by incorporating plasticizers (mainly solvents) to take advantage of both the increased chain mobility of polymers and the ease of dissociation of lithium salts [13, 14].

However, this approach requires a large amount of plasticizer (typically more than 20 wt%) to effectively dissociate lithium salts and crystallized polymer chains and may also induce solvent-related side reactions [15]. Another innovative approach is to align the hopping sites of the polymer chains for lithium-ion transport while minimizing the solvent content. For instance, Yu et al. demonstrated that SPEs composed of lithium bis(trifluoromethanesulfonyl)imide (LiTFSI), and p(VDF-HFP) enhanced the cycle performance of solid-state batteries by mitigating oxygen crossover and Li dendrite penetration [16]. In this case, the hopping of lithium ions was enhanced along with well-oriented fluorinated side groups under ultra-dry conditions (less than 10 wt%). Yang et al. reported the use of well-aligned glucose groups in cellulose films to maximize lithium-ion transport with less than 10 wt% solvent content [17]. Although very high (~ 10^−3^ S cm^−1^) ionic conductivity was achieved using these approaches, time-consuming processes are required to minimize the solvent content or to avoid the formation of crystalline structures of polymer chains by adopting repeated vacuuming processes or immersion in metal ion plasticizers.

Polyrotaxane (PR), a representative structure of mechanically interlocked polymers (MIPs), is formed by threading ring-shaped host molecules (e.g., *α*-cyclodextrin(*α*-CD)) onto linear-shaped guest molecules (e.g., poly(ethylene glycol) (PEG)) [18]. PR has the potential to be applied in lithium batteries owing to its unique structure. Specifically, a single *α*-CD composed of six glucose units contains 18 hydroxyl groups (–OH) that can coordinate with anions and 12 ether oxygen groups (C–O–C) and support lithium-ion transport, thereby facilitating ionic conduction [19]. In addition, threaded *α*-CD molecules on linear PEG provide intrinsically aligned structures of glucose units that is favorable for effective lithium-ion transport. Unfortunately, this structure easily forms a high degree of crystallinity owing to the strong hydrogen bonds between the hydroxyl groups, resulting in the requirement of a large amount of solvent (more than 20 wt%) to mitigate the crystallinity [19]. Furthermore, PR molecules modified to minimize crystallinity generally exhibit hydrophilic properties, which are critical drawbacks in LOBs applications.

In this study, we developed a modified PR-based SPE capable of minimizing the crystallinity and residual solvent through hydrophobic modification of the PR network. The fabricated SPE exhibits high ionic conductivity owing to the alignment of *α*-CD with minimized crystallinity, which promotes lithium ions hopping, and the blockage of water molecule invasion through hydrophobic modification of aligned *α*-CD. The feasibility of developing high-performance solid-state LOBs was examined by combining the SPE and cathode materials.

## Experimental Section

### Fabrication of Solid Polymer Electrolytes

Lithium bis(trifluoromethanesulfonyl)imide (LiTFSI) was dried at 130 °C under vacuum for 12 h before use. The SPEs were fabricated in an argon-filled glove box (H_2_O < 0.1 ppm). First, 0.75 g freeze-dried modified polyrotaxane (mPR), 1 g butyl acrylate, 0.1 g polyethylene glycol diacrylate (PDA), 1 wt% 1-hydroxycyclohexyl phenyl ketone (PI184), and LiTFSI (according to each concentration, with 0.75 being selected as the optimal concentration unless otherwise specified, details in Supplementary Method) were dissolved in 4 mL dimethyl sulfoxide (DMSO) and stirred for 4 h. The resulting homogeneous solution was poured onto a Teflon dish and cured by ultraviolet (UV) light irradiation (365 nm, 9,500 mW cm^−1^) for 10 min. Subsequently, the resulting film was post-cured at 70 °C for 24 h under vacuum. Poly(*α*-cyclodextrin) (PCD)-SPE and PDA-SPE were prepared using the same method as mPR-SPE, but only the mPR in mPR-SPE was replaced with PCD and PDA. (Supplementary Method related to the other polymers are provided in the Supplementary Information).

### Material Characterization

^1^H nuclear magnetic resonance (NMR) spectroscopy (Mercury 400 MHz/CP-MAS) was performed using DMSO-d_6_ as the solvent. X-ray diffraction (XRD) analysis was performed using Cu-K*α* radiation (*λ* = 0.154 nm) on a Rigaku Ultima III diffractometer (2*θ* = 2° − 27°, scan rate = 2° min^−1^ and a step size of 0.04°). Thermogravimetric analysis (TGA) was carried out on TGA Q500 (TA Instrument) from 25 ~ 600 °C at a heating rate of 10 °C min^−1^ in a nitrogen atmosphere. Fourier-transform infrared (FT-IR) spectra were obtained using an Agilent Cary 630 FT-IR spectrometer with attenuated total reflection (ATR), over a range 4000–700 cm^−1^ and a resolution of 4 cm^−1^. Differential scanning calorimetry (DSC) was performed between 80 ~ 200 °C at a rate of 10 °C min^−1^ under nitrogen flow (TA instruments, DSC250). Solid-state ^7^Li NMR spectra were acquired on a 500 MHz Bruker ADVANCE III HD NMR spectrometer using 4 mm CPMAS probes. The ^7^Li NMR spectra were referenced to a 1.0 M LiCl solution at 0 ppm. Spinning speed of 10 kHz, pulse repetition delay of 5 s and *π*/8 pulse were used. Dispersion-corrected density functional theory (DFT-D) simulations were conducted using the Dmol3 module of the Materials Studio program (BIOBIA). The simulation employed a generalized gradient approximation (GGA) with the PBE functional and the DNP basis set version 3.5. The Grimme method was applied to correct the DFT-D, and the orbital occupancy was smeared to 0.05 Ha. The surface morphologies of the electrodes were examined by field-emission scanning electron microscopy (FESEM; SU-70, Hitachi). XRD (Rigaku miniflex 600) operated at 40 kV and 15 mA was employed to test the electrode protection of the SPEs and analyze the changes in the surface composition of the lithium metal.

### Electrochemical Characterization

Characterization of SPEs was conducted using coin cells (2032 type) in an argon-filled glove box (H_2_O < 0.1 ppm). Ionic conductivity was measured using electrochemical impedance spectroscopy (EIS) in the frequency range of 10^6^–1 Hz. A multi-channel electrochemical analyzer (Ivium-n-Stat, Ivium Tech.) was used, and the SPEs were sandwiched between symmetric stainless steel (SS/SPE/SS) cells. The electrochemical stability window was determined by the linear sweep voltammetry (LSV) test at a sweep rate of 10 mV s^−1^ in the Li/SPE/SS cell. The Li^+^ transference number was measured in a symmetric Li/SPE/Li cell. A fixed voltage of 10 mV was applied, and the impedance was measured before and after polarization to correct the polarization resistance.

### In Situ Raman Spectroscopy

Raman spectra were acquired using a real-time confocal microscope (HEDA, WEVE) with a 532 nm excitation laser diode focused onto the electrode surface. The slit width and grating were set to 100 μm and 1200 g mm^−1^, respectively. Each Raman spectrum was collected over a duration of 200 s with two acquisitions and was collected repeatedly every 4 h. In situ Raman spectroscopy was performed using a handmade in situ Raman cell. The detailed cell structure is shown in Fig. S13. The cell assembly is the same as that used for the Swagelok-type cells. For the transmission of Raman signals, we created a small hole in both the polymer electrolyte and lithium foil to position the electrode at the bottom of the cell structure, enabling easy passage for light and Raman signals. The cell assembly was conducted inside an argon-filled glove box (H_2_O < 0.1 ppm). Oxygen (2.0 bar) was introduced into the Raman cell before the in situ Raman analysis. The current and potential data were recorded using an electrochemical analyzer (Ivium-n-Stat, Ivium Tech.).

## Results and Discussion

### Effects of Modification and Cross-Linking: Ion Transport Mechanism by Alignment of α-CDs

**Fig. 1:**
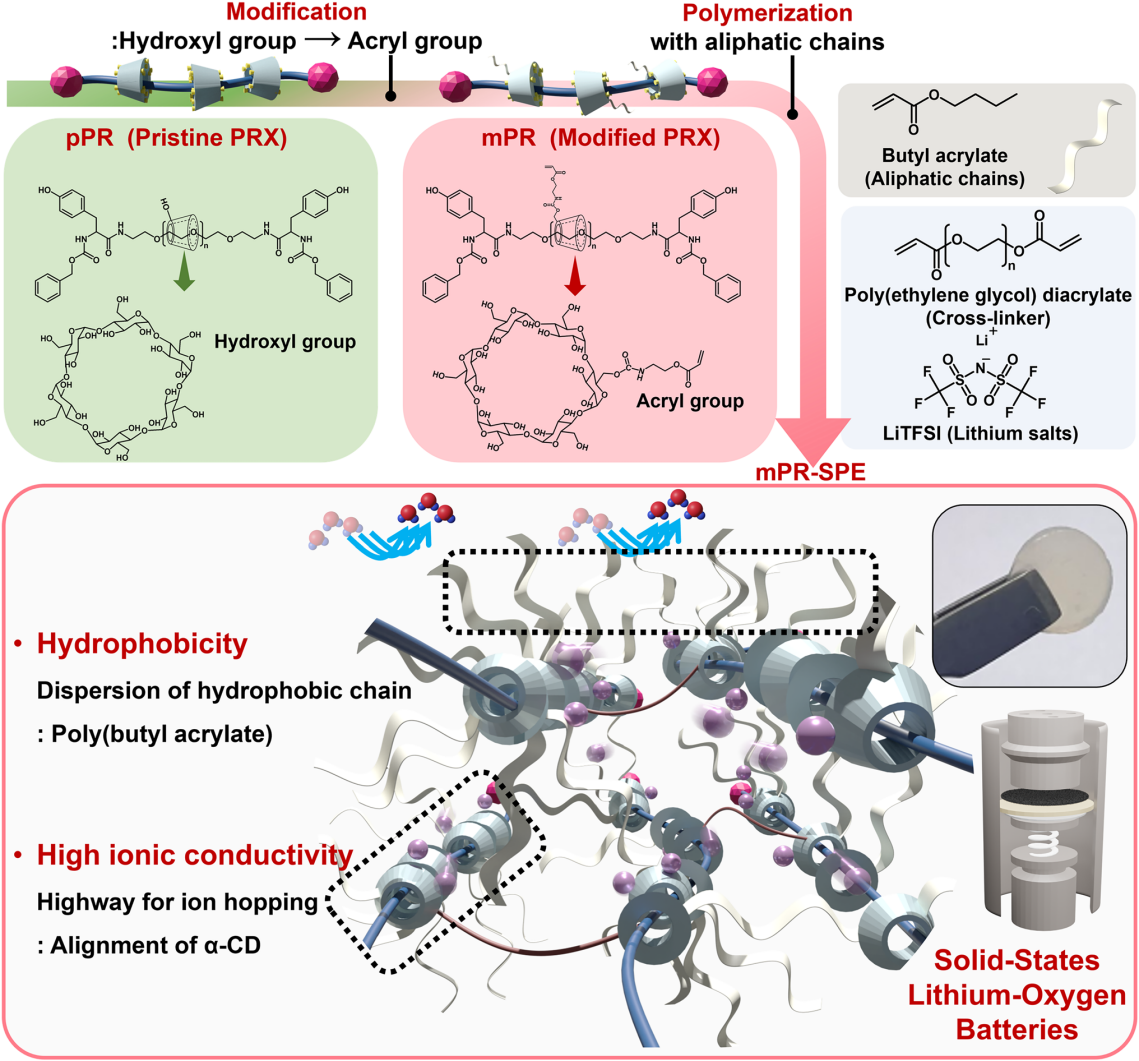
Schematic illustration of the design concept of modified polyrotaxane-based solid polymer electrolyte (mPR-SPE)

**Fig. 2:**
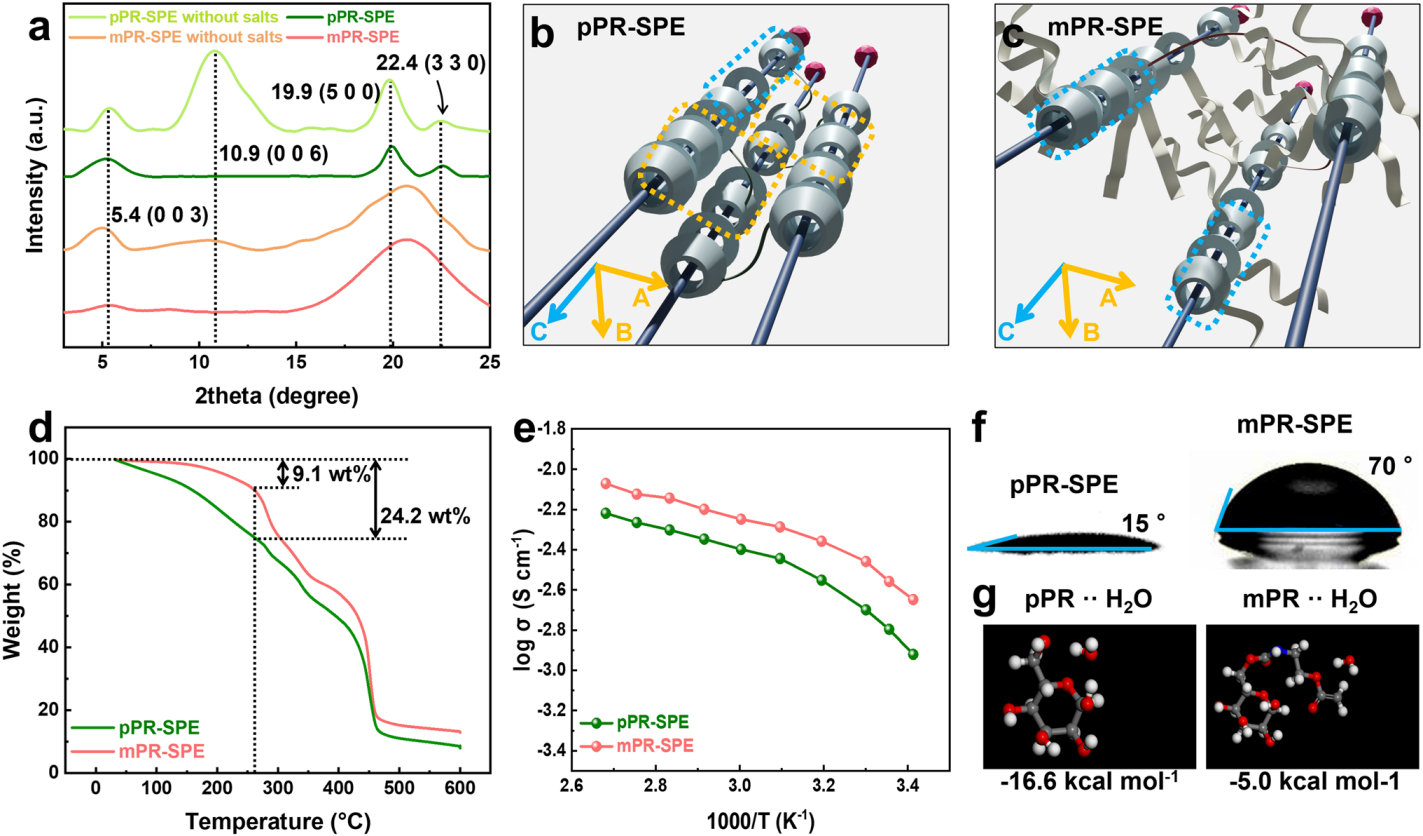
**a** XRD pattern of pPR-SPE, pPR-SPE without salts, mPR-SPE and mPR-SPE without salts. Expected simplified schematic of **b** pPR-SPE and **c** mPR-SPE. **d** TGA data of pPR-SPE and mPR-SPE. **e** Temperature-dependent ionic conductivity of pPR-SPE and mPR-SPE. **f** Water contact angle of pPR-SPE and mPR-SPE. **g** Geometrically optimized three-dimensional (3D) structure of H_2_O and glucose unit of pPR and mPR

The required properties of SPEs for LOB applications include enhanced ionic conductivity. The pristine polyrotaxane (pPR) possesses a structure that facilitates ionic conduction owing to the abundance of hydroxyl and ether-oxygen groups [19]. However, it exhibits a high degree of crystallinity. The crystalline structures of pPR are formed as a result of intermolecular and intramolecular hydrogen bonds between the hydroxyl groups of *α*-CDs [20]. To settle this problem, two main concepts have been adopted, as shown in Fig. 1: modification and cross-linking. The mPR was synthesized by replacing hydroxyl groups with acryl groups. ^1^H NMR (Fig. S1a, b) confirmed that the hydroxyl groups were successfully modified to acryl groups, and the number of acryl groups was estimated to be 1–2. Notably, the XRD results (Fig. S1c) show a decrease in the crystallinity of mPR compared to that of pPR after modification. pPR exhibits a strong crystalline peak at 2*θ* = 7.4°, 12.8°, 19.9°, and 24.4°, corresponding to the plane of PR aggregation. These peaks originate from the hexagonally packed crystalline structure formed by the hydrogen bonding between *α*-CDs [21]. In contrast, mPR exhibits an amorphous halo at 2*θ* = 7.4° and 21°, which corresponds to the short-range order and the facing walls in the single *α*-CD, respectively [22]. The decrease in the formation of the crystalline structure can be attributed to a reduction in the number of hydroxyl groups, which are responsible for crystal formation (Fig. S1d). It is evident that the modification suppressed the formation of a crystalline structure within the material itself. To further confirm the effects of modification and cross-linking on the crystalline structures, mPR and pPR were cross-linked using different methods, both with and without the addition of salt. As shown in Fig. 2a, the cross-linked samples (pPR-SPE and mPR-SPE) exhibit distinct characteristic peaks that differ from those of the non-cross-linked samples (pPR and mPR) (Fig. S1c). The cross-linked samples (pPR-SPE and mPR-SPE) exhibit peak characteristics of the c-axis crystalline structure at 2*θ* = 5.4° and 10.9°, corresponding to the (0 0 3) and (0 0 6) crystallographic planes respectively, while the non-cross-linked sample shows no such peak characteristics [23]. The crystalline structure of PR can be controlled by cross-linking [21, 24]. However, it should be noted that mPR-SPE without salts exclusively exhibits the c-axis crystalline structure (at 2*θ* = 5.4°, 10.9°), whereas pPR-SPE without salts represents the c-axis (at 2*θ* = 5.4°, 10.9°) as well as the a-axis and b-axis (at 2*θ* = 19.9°, 22.4°) crystalline structure. In addition, mPR-SPE exhibits a broad diffraction peak (centered at 2*θ* = 21°), which is typical of an amorphous material [25, 26]. This is because the cross-linking methods of pPR-SPE without salts and mPR-SPE without salts differ because of the structural differences between pPR and mPR. In the case of pPR-SPE without salts, the hydroxyl groups of pPR react with diisocyanate to form a cross-linked network. It is known that in this process, the hydroxyl groups of some *α*-CD do not participate (non-cross-linked) in the reaction, resulting in the formation of a partially cross-linked network [19, 27]. The non-cross-linked *α*-CDs can slide along the PEG, and form crystalline structure oriented to the a, b and c-axis (at 2*θ* = 5.4°, 10.9°, 19.9°, and 22.4°) through intermolecular hydrogen bonding with non-cross-linked or cross-linked *α*-CDs of other pPRs (Figs. 2b, S2). In contrast, in the case of mPR-SPE without salts, a network is formed through radical polymerization between the acryl groups of *α*-CD, butyl acrylate, and PDA. Since all *α*-CDs of mPR possess approximately 1–2 acryl groups (Fig. S1b), non-cross-linked *α*-CDs are not present. As a result, the *α*-CDs align along a single PEG axis rather than interacting intermolecularly with *α*-CDs of other mPRs, leading to the formation of anisotropic crystalline structures oriented along the c-axis through intramolecular hydrogen bonding among the *α*-CDs within each mPR (Figs. 2c, S2). Simultaneously, the presence of poly(butyl acrylate) polymerized between each mPR inhibits intermolecular hydrogen bonding between different mPRs, thereby limiting the possibility of the formation of crystalline structures related to the a and b-axis. These results indicate that after the formation of a polymerizable structure through modification, cross-linking through polymerization can effectively control the alignment of *α*-CDs and the resulting crystalline structure. Following the introduction of LiTFSI, both SPE samples (pPR-SPE and mPR-SPE) exhibit a decrease in the degree of crystallinity owing to the plasticizing effect of LiTFSI [25, 28].

TGA and EIS were conducted to investigate the influence of the residual solvent on ionic conductivity. The residual DMSO content in the SPE samples was estimated by TGA (Fig. 2d). The temperature range of up to 260 °C in the TGA can be attributed to residual DMSO (189 °C, boiling point) [29, 30]. Above 260 °C, the thermal decomposition of the *α*-CD occurs as the first step. The second step corresponds to the decomposition of PEG. The estimated residual DMSO content of mPR-SPE from the TGA curve was approximately 9 wt%, whereas the residual DMSO content of pPR-SPE was approximately 24 wt%. Notably, mPR-SPE exhibited better ionic conductivity than pPR-SPE, despite having a lower residual DMSO (Fig. 2e). In general, the residual solvent plays a crucial role in facilitating the transport of lithium ions within the polymer electrolyte [31, 32]. Furthermore, in the case of the pPR-SPE, DMSO disrupts the hydrogen bonding between *α*-CDs, providing increased mobility of the *α*-CDs and facilitating the transport of lithium ions through the interaction between ether-oxygen groups of the *α*-CDs and lithium ions [19]. However, as mentioned above, since all *α*-CDs of mPR-SPE participate in the cross-linking process and form a network with poly(butyl acrylate), the movement of *α*-CDs is relatively more restricted than that of pPR-SPE. In addition, the alignment of *α*-CD is confirmed by the crystallographic plane of the XRD (Fig. 2a) in the c-axis along the PEG chain. Based on these results, a possible ion transport mechanism that differs from that of pPR-SPE is suggested in Fig. 1. It is presumed that in mPR-SPE, the transport of lithium ions occurs by hopping along the *α*-CDs that are aligned onto the PEG chain, rather than through a shuttle-like movement of the *α*-CDs in pPR-SPE [19]. Thus, the cross-linking through modification and polymerization reduces the dependence of solvent-induced plasticization by alleviating the formation of crystalline structure of the mPR network itself, while also enabling high ionic conductivity through the alignment of *α*-CDs.

### Effects of Modification and Cross-Linking: Electrode Protection Ability

One of the main problems in LOB applications is the vulnerability of lithium metal electrodes to water generated from air. Another effect of the modification and cross-linking of mPR-SPE is enhanced hydrophobicity. pPR is hydrophilic due to the abundance of hydroxyl groups. Therefore, pPR-SPE cannot protect lithium metal electrodes in the operating environment of the cell. For mPR and mPR-SPE, the hydroxyl groups are modified into acryl groups and polymerized with butyl acrylate, which is composed of aliphatic chains. As a result, it is expected that mPR-SPE will exhibit a better protection ability for lithium metal electrodes than pPR-SPE. The water contact angle, shown in Figs. 2f and S3a, represents the degree of hydrophobicity of the SPE sample for each cross-linking method (with PTFE serving as a positive control). Specifically, the contact angles of the pPR-SPE, mPR-SPE, and PTFE were measured to be 15°, 70°, and 108°, respectively. In fact, the lithium ions in lithium salts are hydrophilic, which makes the SPE system sensitive to moisture [33]. Considering this theoretical background, it is reasonable to observe that pPR-SPE exhibits a contact angle of 15°, indicating extremely hydrophilic properties. Nevertheless, mPR-SPE exhibits a contact angle of 70°, which can be attributed to the presence of hydrophobic poly(butyl acrylate) chains distributed throughout the mPR network. These hydrophobic chains created a barrier that reduced the interaction between water and the electrolyte surface, resulting in a higher contact angle. In the case of PTFE, it is a well-known hydrophobic material that exhibits a contact angle of 108°, which is higher than that of other samples.

To further analyze the difference in hydrophobicity, the DFT-D was used to determine the ground state of the molecules and calculate their binding energy [34]. Figures 2g and S3b show the ground state of the water molecules and the unit molecules of each sample, which were obtained through DFT-D simulations. The calculated binding energy between the water molecule and pPR unit was found to be − 16.6 kcal mol^−1^. This was followed by the binding energy between the water molecule and mPR unit (− 5.0 kcal mol^−1^), and the binding energy between the water molecule and PTFE unit (− 2.9 kcal mol^−1^). The DFT-D results clearly show a significant difference in the binding energy between the water molecules and the units of each sample. It is evident that both the contact angle and binding energy exhibit consistent tendencies. In particular, the pPR unit exhibits the strongest binding energy owing to the presence of abundant hydroxyl groups that can form hydrogen bonds with water molecules. This phenomenon indicates that the modification of the acryl group can effectively lower the binding energy between water and the mPR unit, which is the main reason for the improved hydrophobicity. From these results, it is believed that reducing the binding energy through modification and distribution of hydrophobic chains via polymerization within mPR-SPE can effectively enhance its hydrophobicity. The relationship between hydrophobicity and moisture sensitivity was confirmed using the simplified dry cub method, which demonstrates the ability of moisture to penetrate a material [35].

**Fig. 3:**
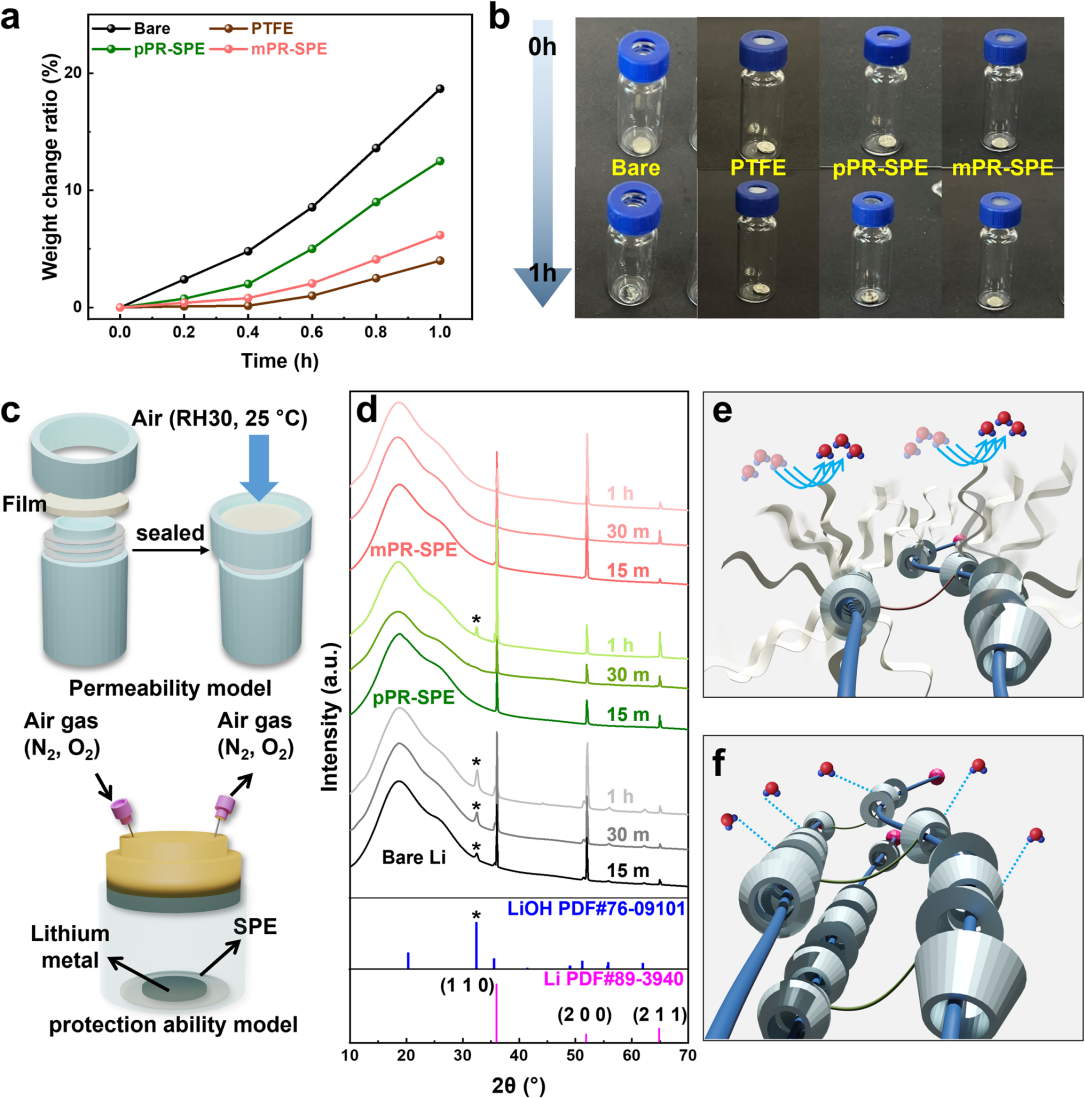
**a** Weight change ratio of each lithium metal over time. **b** Photo images of air permeability model experiment. **c** Schematic diagram of permeability model experiment and protection ability model experiment. **d** XRD results after air protection ability model experiment and expected. Expected interaction with water molecules of **e** mPR-SPE and **f** pPR-SPE

A glass bottle with a specially designed cap is regarded as the “cup” for desiccant. The container was tightly sealed by screwing the cap, with the sample positioned between the cap and glass bottle. This arrangement ensured that humid air could enter the bottle only through the sample (for permeability model). Figure 3a shows the results of the dry cup method over a period of 1 h. After being exposed to humid air (25 °C, 35% RH) for 1 h, the exposed lithium metal in the bare glass bottle undergoes continuous reactions with the moisture in the air (Fig. 3b, c), resulting in a significant increase in weight up to the final value of 18.7%. The pPR-SPE-sealed bottle, on the other hand, exhibits a slightly reduced value of weight increase (12.5%). Clearly different from this, mPR-SPE-sealed bottle and PTFE-sealed bottle exhibit values of 6.2% and 4%, respectively. Notably, mPR-SPE, despite containing hydrophilic salts, effectively impedes the entry of humid air to a similar extent as the PTFE-sealed bottle. To quantitatively confirm this, a protection ability model experiment was conducted using a sealed vial that allowed only air to enter and exit, as shown in Fig. 3c. The lithium metal was completely covered by each SPE, and the air could only contact the lithium metal by passing through each SPE. Figure 3d shows the XRD spectra of the lithium metal surface after exposure to air gas over time. For bare lithium metal, the peak corresponding to LiOH immediately grew upon exposure to the gas, indicating the vulnerability of pure lithium metal to the oxygen and water generated from the air [36]. For pPR-SPE, a peak corresponding to LiOH was observed after approximately 1 h of exposure. By contrast, no obvious peaks corresponding to LiOH were observed for mPR-SPE. This demonstrates that the lithium metal protection ability of mPR-SPE from air was enhanced owing to its better hydrophobicity (Fig. 3e, f). Meanwhile, it is crucial for SPE to effectively prevent the ingress of moisture toward the lithium metal during cell operation. This is because lithium metal undergoes a chemical reaction with moisture, resulting in the rapid degradation of cell performance and posing a safety hazard [37]. Therefore, mPR-SPE suggests that stable cell operation can be achieved by effectively mitigating water penetration into the lithium metal.

### Electrochemical Properties ans Structural Characteristics of mPR-SPE

**Fig. 4:**
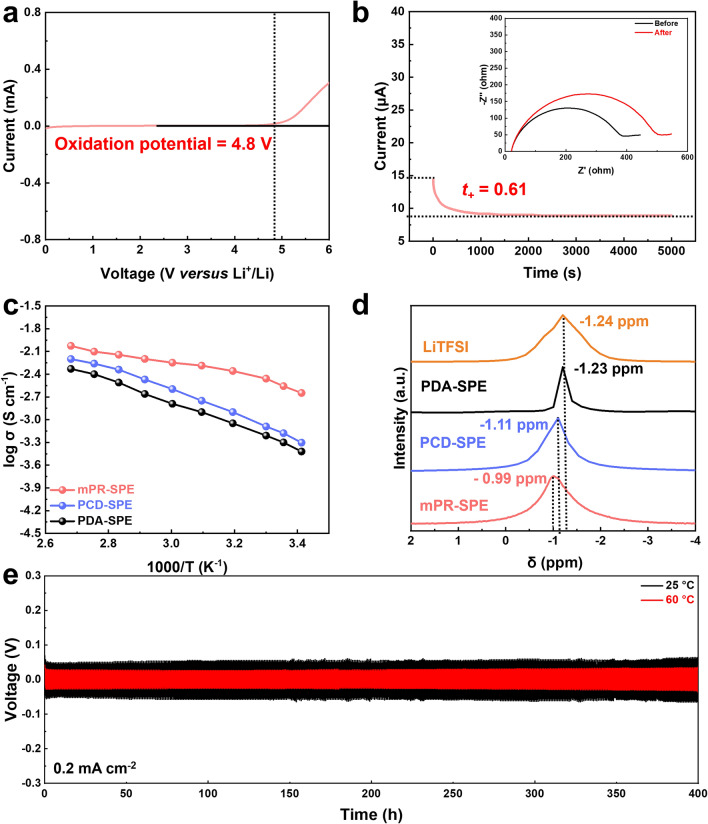
**a** LSV profile of mPR-SPE. **b** Li^+^ transference number of mPR-SPE. **c** Temperature-dependent ionic conductivity of PDA-SPE, PCD-SPE and mPR-SPE. **d** ^7^Li NMR spectra of LiTFSI, PDA-SPE, PCD-SPE and mPR-SPE. **e** Cycling performance of the symmetric Li cells with mPR-SPE

Several other factors must also be considered when developing electrolytes for solid-state LOBs. The mPR-SPE exhibited oxidation stability of up to 4.8 V (vs Li^+^/Li), as shown in Fig. 4a [38, 39]. The lithium transference number (t_+_) was determined using the Bruce–Vincent equation [40], the mPR-SPE exhibited high t_+_ (0.61), which can be attributed to the hopping mechanism of lithium ions along the aligned *α*-CDs (Fig. 4b). The alignment of *α*-CDs in mPR-SPE is crucial for enhancing lithium-ion transport by facilitating hopping of lithium ions. To confirm the effect of *α*-CDs alignment on lithium-ion conduction, various acrylic derivative polymers (Fig. S4a) were prepared for SPE and compared with the necklace-shaped structure of the mPR-SPE. PDA-SPE (linear-shaped) and PCD-SPE (ring-shaped) (Fig. S4b) were prepared. Complete polymerization was confirmed by monitoring the disappearance of the IR bands at 1637 and 815 cm^−1^, which is related to the double bonds of the acrylic monomer after the curing process (Fig. S4c, d). As shown in Figs. 4c and S5a, mPR-SPE exhibits the highest ionic conductivity (2.8 × 10^−3^ S cm^−1^) at 25 °C, while the ionic conductivities of PCD-SPE and PDA-SPE are 6.6 × 10^−4^ and 5.0 × 10^−4^ S cm^−1^, respectively. Ionic conductivity and oxidation stability of the mPR-SPE are compared with those of recently reported polymer electrolytes, as shown in Fig. S6 and Table S1. Additionally, the t_+_ values of the mPR-SPE, PCD-SPE, and PDA SPE were 0.61, 0.39, and 0.32, respectively, which matched the order of ionic conductivity (Figs. 4b, S5b, c). The lithium-ion transport in the linear-shaped PDA was attributed to the segmental motion of the PEG chains. On the other hand, in the ring-shaped structure of PCD, lithium-ion transport occurs through the dangling motion of the *α*-CD pendant groups [19]. Considering the similar acrylic structure and composition of poly(butyl acrylate) within the three structures, the high ionic conductivity observed in mPR-SPE can be attributed to the facilitated hopping of lithium ions along the aligned *α*-CDs. In particular, mPR-SPE exhibits a fivefold higher ionic conductivity than PCD-SPE. This significant difference in ionic conductivity can be attributed to the structural distinction between randomly arranged dangling *α*-CDs in PCD-SPE and the aligned arrangement of *α*-CDs along the PEG axis in mPR-SPE [41].

To investigate the differences in ionic conductivity, the thermal and crystallization behaviors of the samples were separately analyzed for structures with and without salt, enabling a more detailed analysis of the inherent cross-linked structure (Fig. S7). In the case of PDA-SPE, the DSC and XRD spectra indicated its amorphous characteristics, which can be attributed to the typical amorphous nature of PDA [42]. The glass transition temperature (*T*_g_) of PDA-SPE was estimated to be − 47 °C (Fig. S7a and Table S2). However, PDA-SPE exhibited the lowest ionic conductivity. This is because lithium-ion transport depends solely on the segmental motion of the internal polymer chains. On the other hand, the lithium ions inside PCD-SPE and mPR-SPE are generated by hopping along the *α*-CDs in addition to the segmental motion of polymer chains, resulting in higher ionic conductivity than that of PDA-SPE. Notably, PCD-SPE and mPR-SPE exhibit similar thermal behavior with comparable *T*_g_ values of − 44 and − 45 °C, respectively, but they show differences in crystallization behavior. As shown in Fig. S7b, the *α*-CDs in PCD can form the crystalline structure through dangling motion, resulting in the formation of crystalline structures with a, b, and c-axis orientation. This is evident from the peaks observed at 2*θ* = 5.4°, 10.9°, 19.9°, and 22.4°, which correspond to the (0 0 3), (2 $\bar{1}$ 0), (3 0 0), and (5 0 0) planes, respectively. In contrast, in the case of mPR-SPE, the *α*-CDs aligned along the PEG axis owing to the polymerized network, as shown in Fig. 2c. This alignment results in the formation of an anisotropic crystalline structure with c-axis orientation. The peaks observed at 2*θ* = 5.4° and 10.9° correspond to the (0 0 3) and (0 0 6) planes, respectively. Therefore, although they possess similar *T*_g_ and segmental motion, it is believed that the difference in crystallization behavior inherent to each cross-linked structure leads to variations in the lithium-ion hopping pathway and, consequently, differences in ionic conductivity. In all samples, the addition of salt decreased *T*_g_ and increased the amorphous nature due to the plasticizing effect of LiTFSI [25, 28].

Another factor is the difference in ionic conductivity due to structural differences among the SPEs. The presence of dissociated lithium ions was confirmed by ^7^Li NMR spectroscopy, as shown in Fig. 4d. When the lithium ions are in a strongly bound, a peak appears in the high field state because of the large number of electrons formed near the lithium ions. All SPEs (− 1.23 ppm of PDA-SPE, − 1.11 ppm of PCD-SPE, and − 0.99 ppm of mPR-SPE) exhibited a lower field state compared to that of LiTFSI (− 1.24 ppm). For PDA-SPE, no significant shift was observed, due to the lack of dissociation ability within the network. However, both the PCD-SPE and mPR-SPE exhibited lower field shifts than the PDACD-SPE. This is attributed to the interaction between the hydroxyl groups and TFSI^–^ inside the network [19]. It is noteworthy that mPR-SPE exhibited a much lower field than that of PCD-SPE owing to its alignment of *α*-CD. The TFSI^−^ ions inside PCD-SPE are relatively less likely to interact with hydroxyl groups owing to steric hindrance caused by the randomly arranged dangling *α*-CDs [43]. For these reasons, the mPR-SPE exhibits better ion-conduction properties than the other SPEs. The long-term cycle stability of mPR-SPE was investigated during the Li plating/stripping process using symmetric Li/SPE/Li cells at both 25 and 60 °C (Figs. 4e and S8). At a current density of 0.2 mA cm^−2^, the overpotential was ~ 0.06 V at 25 °C and ~ 0.03 V at 60 °C, and no visible short circuit occurred for 400 h.

### Electrochemical Performance of Solid-State Lithium–Oxygen Batteries

**Fig. 5:**
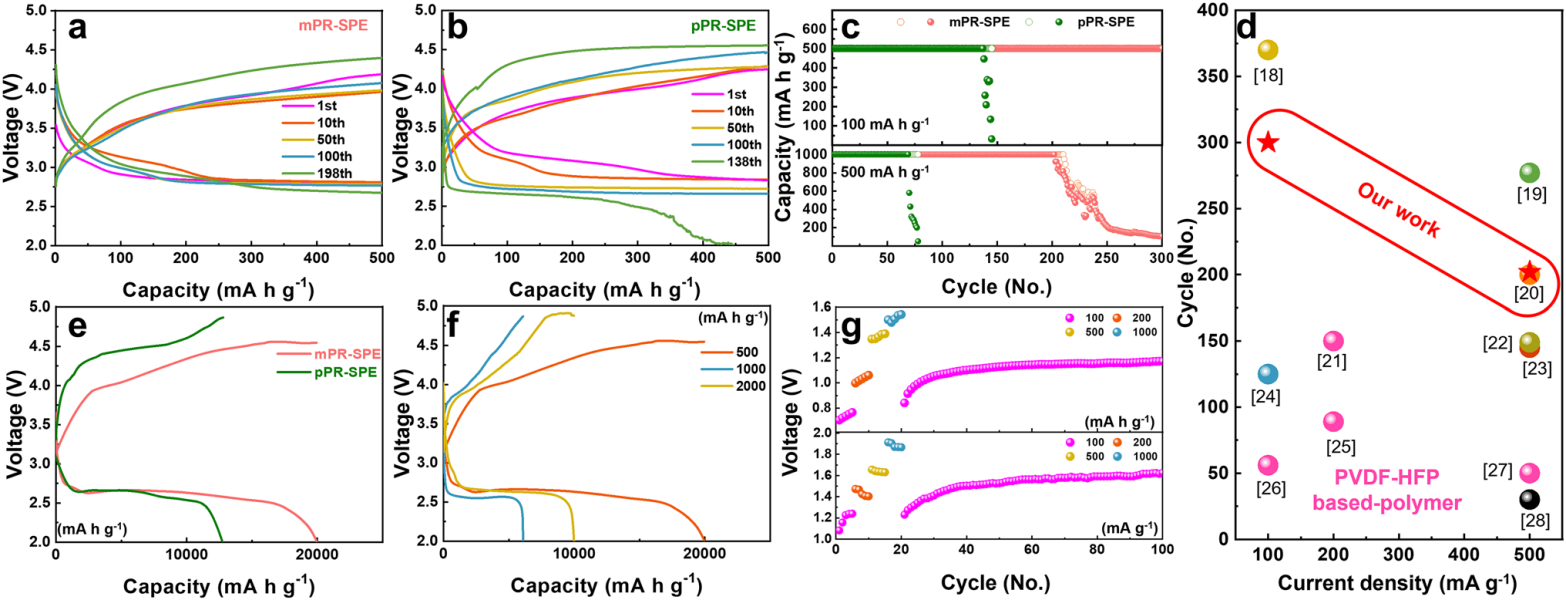
Galvanostatic cycling of solid-state LOBs with **a** mPR-SPE and **b** pPR-SPE at a current density of 100 mA g^−1^ and a fixed capacity limit of 500 mAh g^−1^. **c** Cycling performance of mPR-SPE and pPR-SPE cell associated with Figs. 5a, b and S10. **d** Comparison of the current density and cycle performance with those of previously reported polymer-based solid-state LOBs. **e** Galvanostatic first discharge–charge curves of mPR-SPE and pPR-SPE at a current density of 500 mA g^−1^. **f** Galvanostatic first discharge–charge curves of mPR-SPE cell at different current densities. **g** Overpotential at end potential for each capacity- and current density-controlled cycling corresponding Fig. S12a, b. The controlled analyses of capacity and current density were measured for 5 cycles each, followed by subsequent cycling

To investigate the electrochemical performance of the solid-state LOB applying mPR-SPE, the designed cell was discharge–charge cycled at different temperatures of 25 and 45 °C in the potential range 2.0–5.0 V under an applied current density from 100 to 2000 mA g^−1^. As shown in Fig. S9, a solid-state LOB with a lithium metal anode, SPE, and RuO_2_-graphene (RuO_2_–G) cathode was assembled. The cathodes were fabricated via a simple drop-casting method using RuO_2_–G catalysts on a carbon-fiber film. The loading mass of cathode material (Wcat) was adjusted to 0.45 mg cm^−2^ for reliable comparison. Galvanostatic discharge–charge tests were carried out with a fixed capacity limit of 500 mAh g^−1^ and a current rate of 100 mA g^−1^ (Fig. 5a, b). The cycle curves of the pPR-SPE and mPR-SPE cells were compared for the first, tenth, fiftieth, one-hundredth, and final cycles. During the first cycle, the pPR-SPE and mPR-SPE cells showed charge voltage plateaus of 3.96 and 3.82 V at half-capacity, and low terminal voltage of 3.98 V of mPR-SPE cell whereas the pPR-SPE cell presented 4.28 V, respectively. In particular, only the mPR-SPE cell exhibited stable curves without significant overvoltage changes for up to 50 cycles, indicating good reversibility. In contrast, with an increasing cycle count, the pPR-SPE cell manifested pronounced polarization, marked by a sharp increase in the terminal charge voltage. Upon reaching 138 cycles, the voltage of charge curve for the pPR-SPE cell surpassed 4.5 V, accompanied by a sudden termination of the discharge curve, indicating the end of the cycle life. The mPR-SPE cell demonstrates consistent discharge and charge potentials over 300 cycles, as shown in Fig. 5c. In addition, we measured the galvanostatic discharge and charge profiles of the pPR-SPE and mPR-SPE cells with a current rate of 500 mA g^−1^ and a fixed capacity limit of 1000 mAh g^−1^ (Fig. S10).

The overpotential between the discharge and charge curves in the 1st cycle presents a different voltage profile compared to the subsequent cycles [44]. This phenomenon, previously reported in the literature, is attributed to the initially slow reaction rates for the formation and decomposition of Li_2_O_2_, which are accelerated by catalytic activation in the following cycles. In the 10th cycle, two distinct plateaus are observed in both mPR-SPE and pPR-SPE cells during the discharge process: one at ~ 3.0 V and another at ~ 2.8 V. The first plateau (~ 3.0 V) corresponds to LiO_2_ formation, while the main plateau (~ 2.8 V) is associated with Li_2_O_2_ formation [9, 45]. Other studies have similarly reported the prominent observation of two plateaus during the discharge process when using a solid electrolyte, as opposed to a liquid electrolyte [1, 46]. As cycling progresses, the mPR-SPE and pPR-SPE cells exhibit varying lengths of the LiO_2_ plateau, with the mPR-SPE cell showing a higher capacity associated with LiO_2_ formation than the pPR-SPE cell. This suggests a more intimate contact between the solid electrolyte and electrode, efficient O_2_ absorption, and higher ionic conductivity in the mPR-SPE cell. Conversely, the pPR-SPE cell exhibits a shorter LiO_2_ formation range coupled with a rapid drop in voltage after 60 cycles. These results could be due to side reactions resulting from the formation of O_2_^−^ dissolved in the remaining 24% DMSO solvent during the charge/discharge process inside the pPR-SPE rather than the formation of LiO_2_ on the surface. Consequently, the combination of excellent ionic conductivity and a low concentration of residual solvent in the mPR-SPE cell demonstrates superior characteristics of low overpotentials and stable cycling performance over extended cycles (210 cycles).

The properties (current density, capacity, cycle performance, and associated cell components) of the prepared solid-state LOBs are compared with those of recently reported polymer electrolytes, as shown in Fig. 5d and Table S3. The mPR-SPE cell exhibits excellent performance even without the addition of fillers or plasticizers to the electrolyte. Figure 5e presents the 1st galvanostatic discharge–charge curve of two SPE cells at a current rate of 500 mA g^−1^. The charge capacities of the pPR-SPE and mPR-SPE cells are limited to the same discharge capacity. The mPR-SPE cell delivers higher discharge capacities than that of pPR-SPE cell (20,000 versus 12,700 mAh g^−1^, respectively) and the overall overpotential during charge/discharge is significantly lower in the mPR-SPE cell.

To assess the feasibility of solid-state LOB under elevated temperatures, we examined cell performance at 45 °C with a current density of 500 mA g^−1^. As shown in Fig. S11, the cells tested at high temperatures exhibit notably higher overall overpotential than those tested at 25 °C. The temperature elevation also introduces instability into the battery system. First, increased temperatures concurrently promote parasitic reactions, leading to the formation of Li_2_CO_3_ or LiOH by-products, as evidenced by the rapid voltage spikes and cut-off voltage drops in cells that utilize pPR-SPE. Additionally, elevated temperatures exacerbate electrolyte volatilization, resulting in an increased concentration or precipitation of oxidized lithium salts, potentially obstructing cathode pores and causing abrupt discharge termination [47]. This phenomenon was more pronounced in the pPR-SPE cell with a higher residual solvent. The pPR-SPE cell demonstrates an extended cycle life of 98 cycles at high temperatures compared to the 25 °C (69 cycles). Conversely, mPR-SPE exhibits a diminished lifespan from 200 to 135 cycles under similar conditions. This discrepancy is attributed to the different portions of the internal residual solvent in the polymer network. It is possible that the faster ion/charge transfer at high temperatures is due to the high residual solvent of pPR-SPE (24 wt%), resulting in a longer lifespan. In contrast, mPR-SPE is relatively sensitive to temperature because its solvent content is as low as 9 wt%. Nevertheless, it is notable that the mPR-SPE cell exhibits superior cycling stability compared to the pPR-SPE cell, even at high temperatures. As shown in Fig. 5f, at higher applied current densities from 500 to 2000 mA g^−1^, the cell containing the mPR-SPE still exhibited an acceptable range of discharge capacity from 20,000 to 6000 mAh g^−1^, as well as the gradual decrease in the discharge potential plateau from 2.62 to 2.55 V. Up to a current density of 2000 mA g^−1^, the overpotential between the discharge and charge potentials gradually widens. Increased current density expanded the overpotential gap at half capacity (1.77 V at 1000 mA g^−1^ and 1.79 V at 2000 mA g^−1^). The cycle performance of the mPR-SPE cell at different capacity limits (100, 200, 500, and 1000 mAh g^−1^) was also estimated as shown in Fig. S12a. Each different capacity-limited curve was evaluated for 5 cycles, after which the cycle was evaluated with a capacity limit of 100 mAh g^−1^ (Fig. 5g). As the limited capacity increased, the overpotential at the end cpacity increased (0.70 V at 100 mAh g^−1^, 1.0 V at 200 mA g^−1^, 1.34 V at 500 mAh g^−1^, 1.50 V at 1000 mAh g^−1^, respectively). It can be observed that the voltage gap does not change significantly. When returning to the cycle of capacity-limited of 100 mAh g^−1^, it exhibits a similar overpotential difference to the initial capacity limit of 100 mAh g^−1^ (0.84 V), but gradually increased to 1.16 V until 100 cycles. The mPR-SPE cells show reliable stability at different capacity limits, maintaining a low charging end potential below 4.5 V at a current density of 1000 mA g^−1^. A key aspect of the practical application of solid-state LOBs is the favorable rate capability of the cells. The rate capability of the mPR-SPE cell in LOBs was also investigated across various current densities ranging from 100 to 1000 mA g^−1^ (Fig. S12b). The overpotential at the end capacity at different current densities, as illustrated in Fig. 5g, was measured over five cycles at each rate, and the corresponding end-capacity overpotential were calculated. With increasing current density, the overpotential between the discharge and charge plateaus (end of discharge capacity) rose from 1.08 V (100 mA g^−1^) to 1.92 V (1000 mA g^−1^). Notably, the voltage gap at a current density of 100 mA g^−1^ gradually increased over 5 cycles. This is attributed to the initially slower rate of Li_2_O_2_ formation and decomposition, which accelerated due to the catalytic activation in subsequent cycles [48]. However, contrary to the trend observed in previous cycle analyses of capacity limitations (Fig. 5f), the initial overpotential exceeded that of the subsequent cycles, particularly at current densities of 200, 500, and 1000 mA g^−1^ during battery cycling. This is because higher current densities result in greater nucleation driving forces, leading to denser but smaller particles, thereby increasing polarization [49]. Notably, this trend was not observed at a current density of 100 mA g^−1^, suggesting it as the optimal current density. These findings are consistent with previous results, where cycles analyzed at a current density of 100 mA g^−1^ exhibited superior stability (300 cycles) compared to those analyzed at 500 mA g^−1^ (202 cycles) in the galvanostatic cycle curves. Consequently, the mPR-SPE cell demonstrates an exceptionally low overpotential, cycle stability, and rate capability across a wide range of current densities. These phenomena may be attributed to three possible factors: (1) the unique structure of the mPR-SPE, characterized by alignment of *α*-CDs, induces high ionic conductivity (2.8 × 10^−3^ S cm^−1^). This facilitates rapid lithium-ion transport, effectively promoting the formation and decomposition of Li_2_O_2_ and thereby significantly reducing polarization. (2) By leveraging the hydrophobic chains within the polymer network, the permeation of moisture and oxygen is efficiently impeded. Suppressing sub-reactions, such as the formation of LiOH on the lithium surface, ensures the overall lifespan of solid-state cells. (3) With a relatively low solvent content of 9 wt% in the polymer electrolyte, the effects of solvent volatilization in high-pressure oxygen environments and side reactions induced by active oxygen species are minimal. Consequently, the combination of high ionic conductivity, hydrophobic properties, and low residual solvent content of mPR-SPE results in low overpotential characteristics with stability, even at high current rates during continuous cycling.

### Detailed Reaction Kinetics During Electrochemical Cycling

**Fig. 6:**
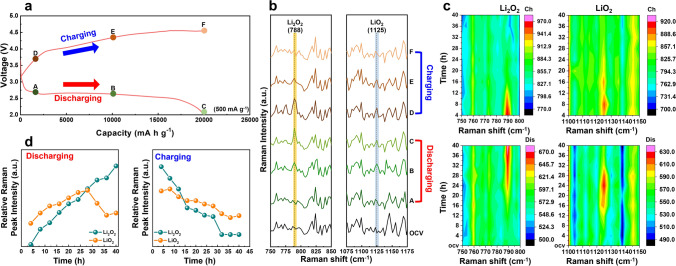
**a** First galvanostatic discharge–charge curves of mPR-SPE cell at a current rate of 500 mA g^−1^. **b** In situ Raman spectra of mPR-SPE cell from the discharge to the charge process recorded at different stages corresponding to Fig. 6a. **c** Contour profile plot or the Raman spectra of the mPR-SPE cell from the discharge to the charge process. **d** Relative Raman peak intensities as a function of time during the discharging and charging process

In situ electrochemical Raman spectroscopy data provided direct evidence of the chemical species at the interface between the polymer electrolyte and the oxygen electrode, offering insights into the O_2_ oxidation/reduction mechanism in solid-state LOB (Fig. 6). To analyze the reactions that occur at the interface between the electrolyte and the electrode using in situ Raman analysis, we adopted a structure with small holes in the electrolyte and lithium metal to allow the laser to pass through to the sample (Fig. S13). In addition, to implement a carbon fiber film as an oxygen electrode environment closely associated with the polymer electrolyte, we infiltrated the polymer electrolyte onto the electrode to form a thin and uniform polymer layer on the electrode surface. The Raman laser focus was positioned between the polymer and the electrode for detailed analysis. The first discharge–charge curves at various points (marked by colored balls) were employed to determine the mechanism of the oxygen reaction of the mPR-SPE cell. Figure 6a illustrates the discharge–charge curves spanning a voltage range of 2.0–5.0 V and a current density of 500 mA g^−1^. The mPR-SPE cell exhibits a high capacity of 20,000 mA h g^−1^ with an overpotential of 1.69 V at half capacity. Figure 6b shows the Raman spectra of the cathodes at various stages of charging and discharging, including after 4, 20, and 40 h. Notably, two distinct peaks are observed at 788 and 1125 cm^−1^, corresponding to the formation of Li_2_O_2_ and LiO_2_, respectively, during the discharge process up to 40 h as discharge products in our LOB (Figs. 6c and S14) [50]. In the initial discharge stage (indicated by the green ball A at 4 h), faint peaks of Li_2_O_2_ and LiO_2_ were observed. Initially, O_2_ was reduced to LiO_2_ via a one-electron transfer. LiO_2_ acts as an intermediate in the formation of Li_2_O_2_, which is primarily localized at the interface between the polymer electrolyte and the oxygen electrode. It reacts with lithium ions to yield Li_2_O_2_. However, the subsequent disproportionation of LiO_2_ in the electrolyte is not expected in solid-state LOB because of the absence of LiO_2_ ion pairs resulting from strong lithium ion solvation in the solid-state electrolyte. Therefore, the formation of Li_2_O_2_ via the uptake of lithium ions and additional electrons via the surface pathway is more reasonable under these cell conditions [46, 51, 52]. Enhancing the uniform formation of LiO_2_ and Li_2_O_2_ along the intimate interface can enhance the reversibility and efficiency of solid-state LOB by mitigating oxidation/reduction overpotentials [53]. As the discharge progresses, the discharge voltage decreases, accompanied by an increase in the intensity of the Li_2_O_2_ peak, indicating a continuous reduction to Li_2_O_2_ (marked by green ball B, 20 h) (Fig. 6d). However, by the end of the discharge phase (marked by the green ball C, 40 h), LiO_2_ is no longer observed on the surface. As Li_2_O_2_ accumulates on the electrode, the available surface for O_2_ adsorption diminishes, thereby inhibiting the sustained formation of LiO_2_ over time. Therefore, a gradual decrease in the intensity of the LiO_2_ peak is observed [46]. At lower potentials (below 2.5 V), this result indicates a significant enhancement in the conversion rate of LiO_2_ to Li_2_O_2_. The LiO_2_ is primarily converted into Li_2_O_2_ through an electron-transfer reaction with Li^+^ ions. Lower potentials result in higher surface concentrations of LiO_2_, promoting further reduction and increasing the overpotential, thereby driving the rate of the second reduction [46].

The charging process comprises three stages. In the initial stage (marked by the brown ball D, 4 h), a plateau was formed at approximately 4 V, with simultaneous peaks of Li_2_O_2_ and LiO_2_ products in the Raman spectrum. The rapid decrease in the Li_2_O_2_ peak intensity and the presence of the LiO_2_ peak during the initial charging suggested the concurrent oxidation of Li_2_O_2_ and formation of LiO_2_. In the second stage (marked by the brown ball E, 20 h), a slight decrease in the Li_2_O_2_ peak intensity was observed, while the LiO_2_ peak ratio steadily decreased. These two steps during charging exhibited curves with different slopes, indicating the presence of two distinct oxidation stages. The abrupt increase in the potential during the initial charging process (step D) signifies a reaction in which lithium ions are extracted from the Li_2_O_2_ surface to form LiO_2_. Subsequently, the process includes the decomposition of LiO_2_ into lithium ions and O_2_ (Step E). The decomposition of Li_2_O_2_ remains unclear and focuses on a controversial topic regarding the locations of the reactions involved: both the electrode/Li_2_O_2_ interface and the electrolyte/Li_2_O_2_ interface are considered possible. Considering the two aforementioned oxidation reaction sites, it is inferred that in the solid-state LOB using mPR-SPE, the reactions primarily occur at the electrolyte/Li_2_O_2_ interface. This is attributed to the ease of oxidation facilitated by the extraction of lithium ions from the collapsed electrolyte/Li_2_O_2_ interface, resulting in a diminished increase in the overpotential [54]. Additionally, if a reaction occurred at the electrode/Li_2_O_2_ interface, the overpotential increase owing to continuous contact shortening could not be ignored, and a rapid overpotential increase in the second stage would have been confirmed. In the final charging stage (marked by the brown ball F, 40 h), neither Li_2_O_2_ nor LiO_2_ peaks were detected. During electrochemical decomposition, LiO_2_ serves as an intermediate species between the oxidation and reduction of Li_2_O_2_, but it is not detectable above 4.5 V [55]. These results indicate the effective oxidation of Li_2_O_2_ decomposition into lithium ions and oxygen when using mPR-SPE. It is noteworthy that the Raman spectra of all cathodes up to 40 h of discharging and charging do not exhibit peaks corresponding to other lithium-based species, such as Li_2_CO_3_ (1093 cm^−1^) or LiOH (858 cm^−1^) [56, 57]. These results demonstrate the electrochemical stability and reversibility of the oxygen reaction. Furthermore, the Raman spectra of the oxygen electrode indicate that the Li_2_O_2_ peaks at 788 cm^−1^ disappeared after the charge process, suggesting reversible formation and decomposition of Li_2_O_2_ nanoparticles in our solid-state LOB. Discharge–charge mechanism was proposed for solid-state LOB employing mPR-SPE, which was elucidated through integrated Raman spectral analysis. The high ionic conductivity of the mPR-SPE facilitates efficient ion transfer to the electrolyte–electrode interface, promoting the formation of LiO_2_ intermediates. These intermediates undergo a second one-electron transfer electrochemical process or disproportionation reaction, leading to the formation of Li_2_O_2_ at the interface. Additionally, the low residual solvent content in the polymer electrolyte enhances the cycle stability by inhibiting the formation of side products, such as LiOH and Li_2_CO_3_, thus reducing the overpotential in solid-state LOB. The novel mPR-SPE, in which reduction of crystallinity and alignment of ion conduction pathway is achieved through modification and polymerization, actively participates in reversible oxygen reaction, making it suitable for solid-state LOBs.

## Conclusions

Our study addressed the critical challenges in the development of solid-state lithium-oxygen batteries (LOBs) caused by inevitable problems with liquid electrolytes (LE). To address the limitations of LEs while aiming to enhance the conductivity of solid polymer electrolytes (SPEs), we presented a novel approach using modified-polyrotaxane (mPR)-based SPE. Modification and polymerization with acryl groups aligned the *α*-cyclodextrin (*α*-CD) inside the mPR, creating a pathway for ion conduction, thereby reducing the influence of solvents and resulting in high ion conductivity. Its designed structure enables efficient lithium-ion transport, while its hydrophobic nature effectively prevents moisture ingress and mitigates side reactions at the lithium–metal interface. Benefiting from these advantages, the solid-state LOB exhibit high discharge capacity, superior rate performance, and extended cycle life. Additionally, in situ Raman spectroscopy revealed the involvement of the LiO_2_ intermediate in the formation of Li_2_O_2_, with no detectable side reaction. Our findings demonstrate the feasibility of improving the ionic conductivity without compromising safety and stability, offering a potential solution to advance solid-state LOB technology. This work contributes to the ongoing efforts to overcome key barriers to the commercialization of high-performance lithium-oxygen batteries for sustainable energy-storage applications.

## Supplementary Information

Below is the link to the electronic supplementary material.

Supplementary file1 (DOCX 5000 KB)
