# Supplementary material for: Aligned Ion Conduction Pathway of Polyrotaxane-Based Electrolyte with Dispersed Hydrophobic Chains for Solid-State Lithium–Oxygen Batteries
Source: Nanomicro Lett. 2024 Oct 1;17:31. doi: 10.1007/s40820-024-01535-w (PMC11445217; doi:10.1007/s40820-024-01535-w)
Supplement: Supplementary file 1 — Supplementary file1 (DOCX 5000 KB) [file 40820_2024_1535_MOESM1_ESM.docx]

Supporting Information for

**Aligned Ion Conduction Pathway of Polyrotaxane-Based Electrolyte with Dispersed Hydrophobic Chains for Solid-State Lithium-Oxygen Batteries**

Bitgaram Kim^1, #^, Myeong-Chang Sung^2, #^, Gwang-Hee Lee^3, #^, Byoungjoon Hwang^2^, Sojung Seo^1^, Ji-Hun Seo^1,^ *, and Dong-Wan Kim^2,^ *

^1^Department of Materials Science and Engineering, Korea University, 145 Anam-ro, Seongbuk-gu Seoul, 02841, Republic of Korea

^2^School of Civil, Environmental and Architectural Engineering, Korea University, 145 Anam-Ro, Seongbuk-gu, Seoul, 02841, Republic of Korea

^3^Materials Science and Chemical Engineering Center, Institute for Advanced Engineering (IAE), Yongin 17180, Republic of Korea

^#^Bitgaram Kim, Myeong-Chang Sung, Gwang-Hee Lee have contributed equally to this work.

*Corresponding authors. E-mail: [seojh79@korea.ac.kr](mailto:seojh79@korea.ac.kr) (Ji-Hun Seo); [dwkim1@korea.ac.kr](mailto:dwkim1@korea.ac.kr) (Dong-Wan Kim)

**S1 Supplementary Method**

**S1. 1 Materials**

Poly(ethylene glycol) (Mn = 10,000), dimethyl sulfoxide (DMSO) (anhydrous, ≥ 99.9%), N,N-dimethylformamide (DMF) (anhydrous, 99.8%), chloroform (anhydrous, ≥99%), butyl acrylate (≥99%), poly(ethylene glycol) methacrylate (PEGMA, Mn=500), 2,2’-azobis(2-methylpropionitrile) (AIBN) and 1-hydroxycyclohexyl phenyl ketone (PI184, 99%) were purchased from Sigma-Aldrich. 1,1’-carbonyldiimidazole (CDI), α-cyclodextrin (α-CD), polyethylene glycol diacrylate (PDA, n=approx.9), ethylenediamine, 4-(4,6-dimethoxy-1,3,5-triazin-2-yl)-4-methylmorpholinium chloride (DMTMM) and N-carbobenzoxy-L-tyrosine (Z-Try-OH), dibutyltin dilaurate (DBTDL, >95%), hexamethylene diisocyanate (HDI), 4,4’-methylene diphyenyl diisocyanate (MDI) were purchased from TCI. Lithium bis(trifluoromethanesulfonyl)imide (LiTFSI, 98+%) was purchased from Alfa Aesar. 2-isocyanatoethyl acrylate was purchased from Showa Chemical Industry. All other organic solvents (HPLC grade) were purchased from Samchun Pure Chemical Co., Ltd. (Gyeonggi-Do, Korea). All chemicals were used as received without further purification.

**S1.2 Synthesis of pristine polyrotaxane (pPR) and pPR-SPE**

2 g poly (ethylene glycol) was completely dissolved in 30 ml chloroform. Subsequently, 0.2 g 1,1’-carbonyldiimidazole was added and stirred for 6 h. Following that, ethylene diamine was added, and the solution was stirred for 24 h. The resulting solution was precipitated in diethyl ether to obtain PEG-bis(amine) (PEGBA) powder. 3 g of α-CD, and 1 g of PEGBA were dissolved in 35 mL of distilled water and allowed to mix for 24 h. The resulting mixture was freeze-dried and dispersed in ethanol (35 mL). Subsequently, DMTMM (0.5 g) and Z-Try-OH (0.5 g) were added and the mixture was stirred vigorously for 24 h. The resulting solution was placed in a dialysis tube (MWCO 12k) and dialyzed, and freeze-dried to obtain pPR powder. pPR-SPE was synsthesized as described in a previous paper[1]. Speficially, 0.6 pPR were disolvend in 6 mL of DMSO with 0.1 M cross-linker (HDI:MDI= 4:1, molar ratio). Then, 0.75 M LiTFSI was added to the solution and stirred until the mixture was completely dissolved. The resultant solution was poured into a PTFE mold within argon-filled glass case ,and reacted in the oven at 70 °C for 48 h.

**S1.3 Synthesis of modified polyrotaxane (mPR)**

1 g pPR, 2-isocyanatoethyl acrylate and 0.1 g DBTDL were dissolved in 6 mL of DMSO. The mixture was stirred vigorously for 36 h. The resulting solution was placed in a dialysis tube (MWCO 12k), dialyzed, and freeze-dried to obtain the mPR powder.

**S1.4 Synthesis of poly(α-cyclodextrin) (PCD)**

1 g PEGMA was dissolved in 6 mL of chloroform and added in a solution in which 0.7 g of 1,4-phenylene diisocyanate was dissolved in 9 mL of chloroform, and resulting solution was stirred for 16 h. After the reaction, the mixture was precipitated in hexane and brown isocyanate-terminated PEGMA (I-PEGMA) was obtained. The collected I-PEGMA and 1 g of α-CD were dissolved in 10 mL of DMF and stirred for 24 h. The resulting solution was then precipitated in diethyl ether to obtain the resultant (CD-PEGMA). Subsequently, 1 g of CD-PEGMA and 0.3 mL AIBN were stirred in 8 mL of DMF and polymerized at 70 °C for 24 h. The solution was then precipitated in diethyl ether. Finally, a brown powder (PCD) was obtained and dried under vacuum.

**S1.5 Optimization of lithium salt concentration of mPR-SPE**


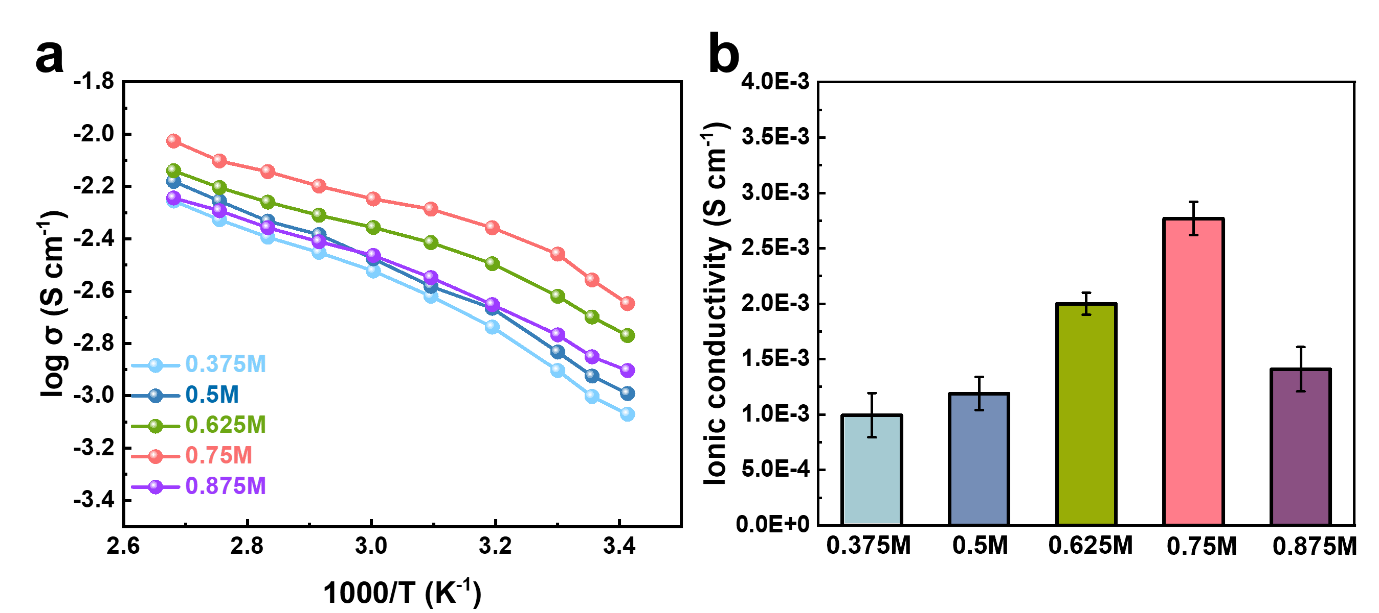


**Extended Fig. 1** (**a**) Temperature-dependent ionic conductivity of the mPR-SPE at LiTFSI concentration of 0.375M, 0.5M, 0.625M, 0.75M, and 0.875M. (**b**) Bar graph of ionic conductivity of the mPR-SPEs at room temperature

Ionic conductivities were investigated to determine the optimal salt concentration (0.75 M) required in mPR-SPE.

**S1.6 Preparation of RuO2-Graphene (RuO_2_-G)**

To synthesize RuO2-G, graphene (0.02 g, Angstron Materials), ruthenium (III) chloride (RuCl3·xH2O; 0.015 g, Kojima Chemical, 99%), and cetrimonium bromide (1 g, Sigma Aldrich, 95%) were dispersed in 25 mL of distilled water and ethanol (4:1, v/v). The resulting solution was sealed in a 30 mL Teflon-lined stainless-steel autoclave and heated at 150 °C for 10 h. After cooling to room temperature, the particles were washed with distilled water, filtered multiple times using a nylon membrane (Durapore, 0.22 μm, Billerica, MA, USA), and dried at 70 °C for 24 h. Subsequently, the particles were subjected to thermal treatment at 500 °C for 2 h, followed by 800 °C for 5 h under a nitrogen flow of 100 sccm.

**S2 Supplementary Figures and Tables**


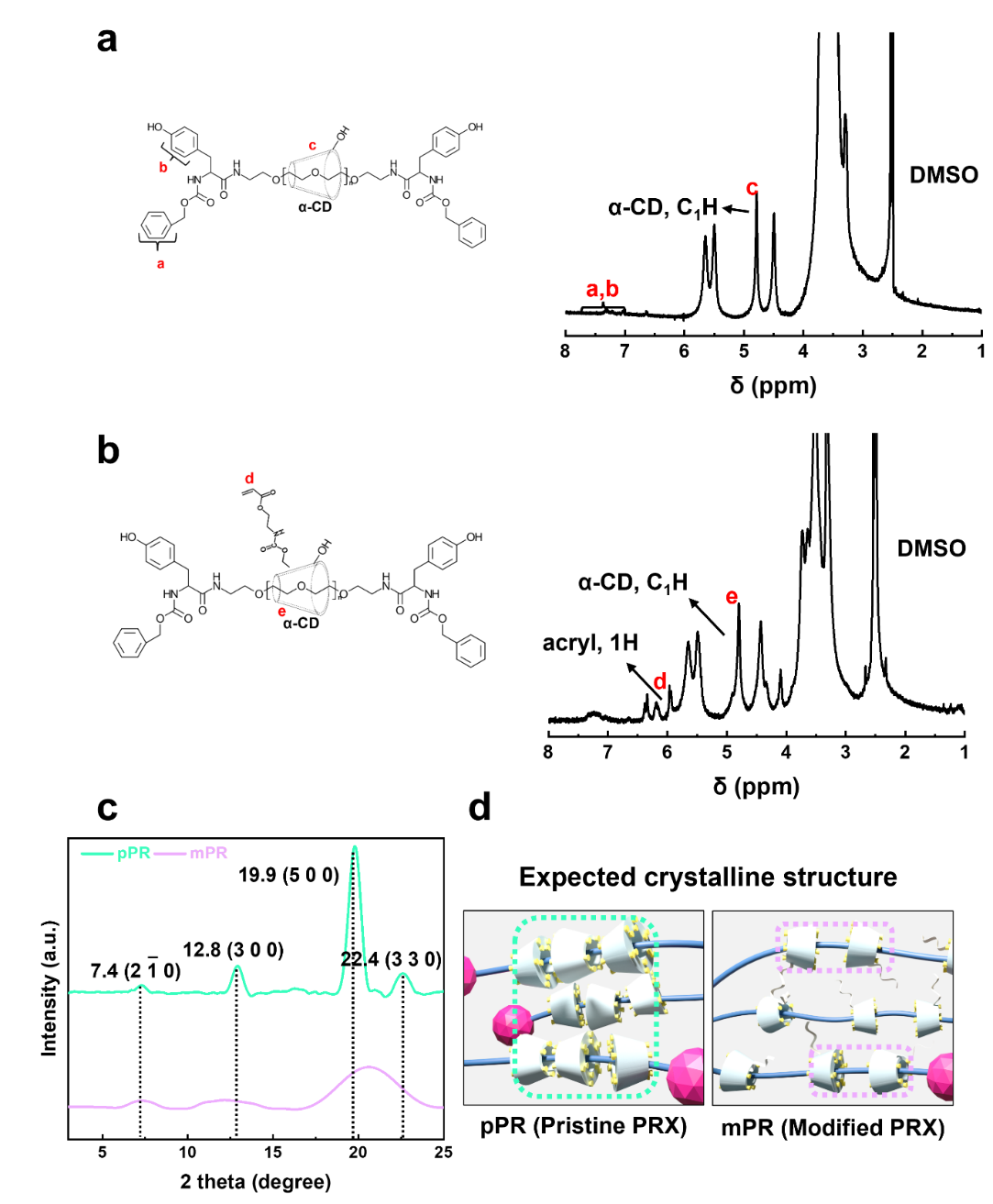


**Fig. S1** (**a**) ^1^H NMR spectrum of pPR (400 MHz, DMSO-D_6_). (**b**) ^1^H NMR spectrum of mPR (400 MHz, DMSO-D_6_). (**c**) X-ray diffraction (XRD) of pPR and mPR. (**d**) Expected crystalline structure of mPR-SPE (In the view of one side of hexagonal lattice)


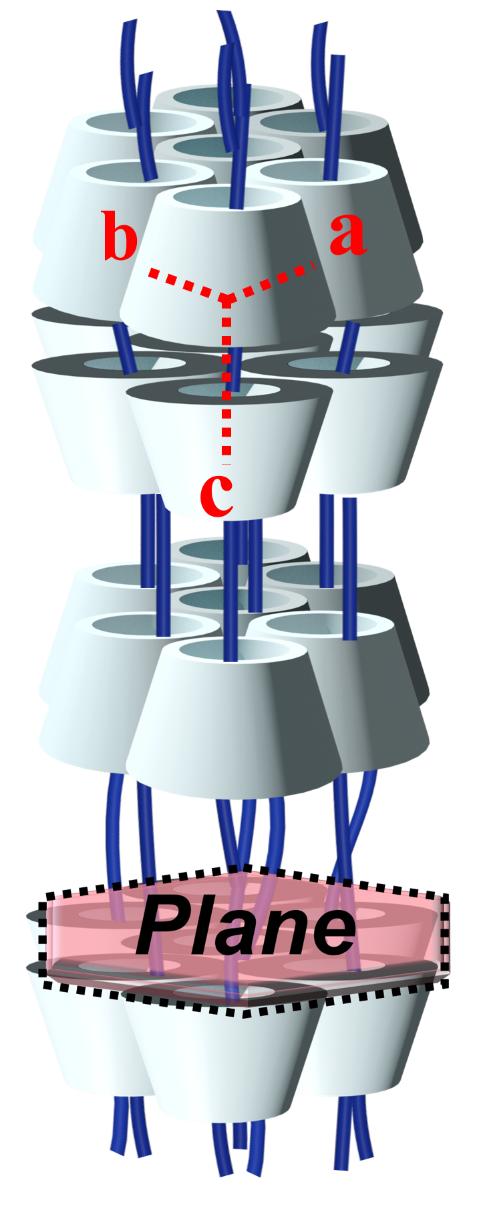


**Fig. S2** Schematic of the crystalline structure by α-CD in polyrotaxane (PR)


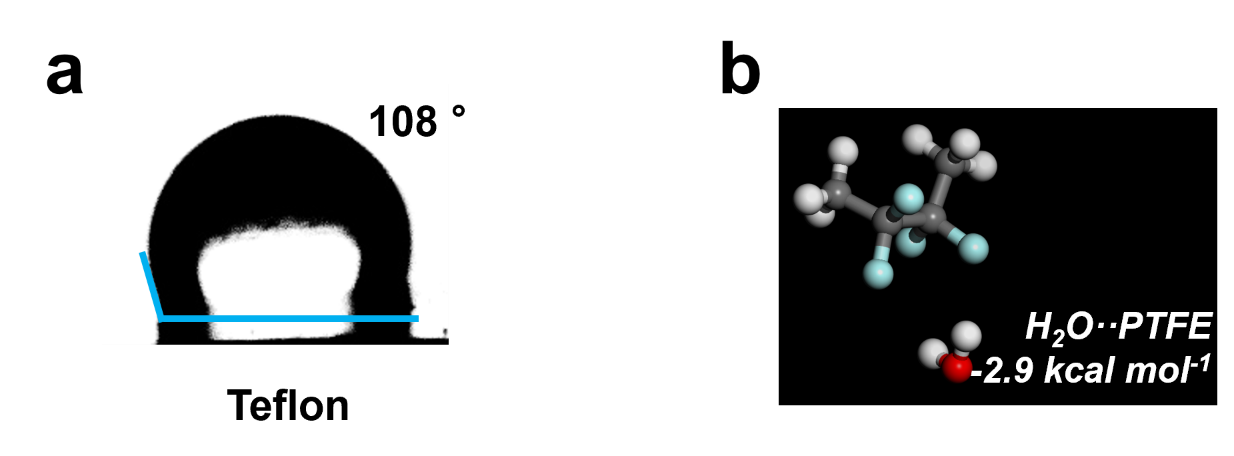


**Fig. S3** (**a**) Water contact angle of Teflon. (**b**) Geometrically optimized three-dimensional (3D) structure of H_2_O and PEFE unit of Teflon


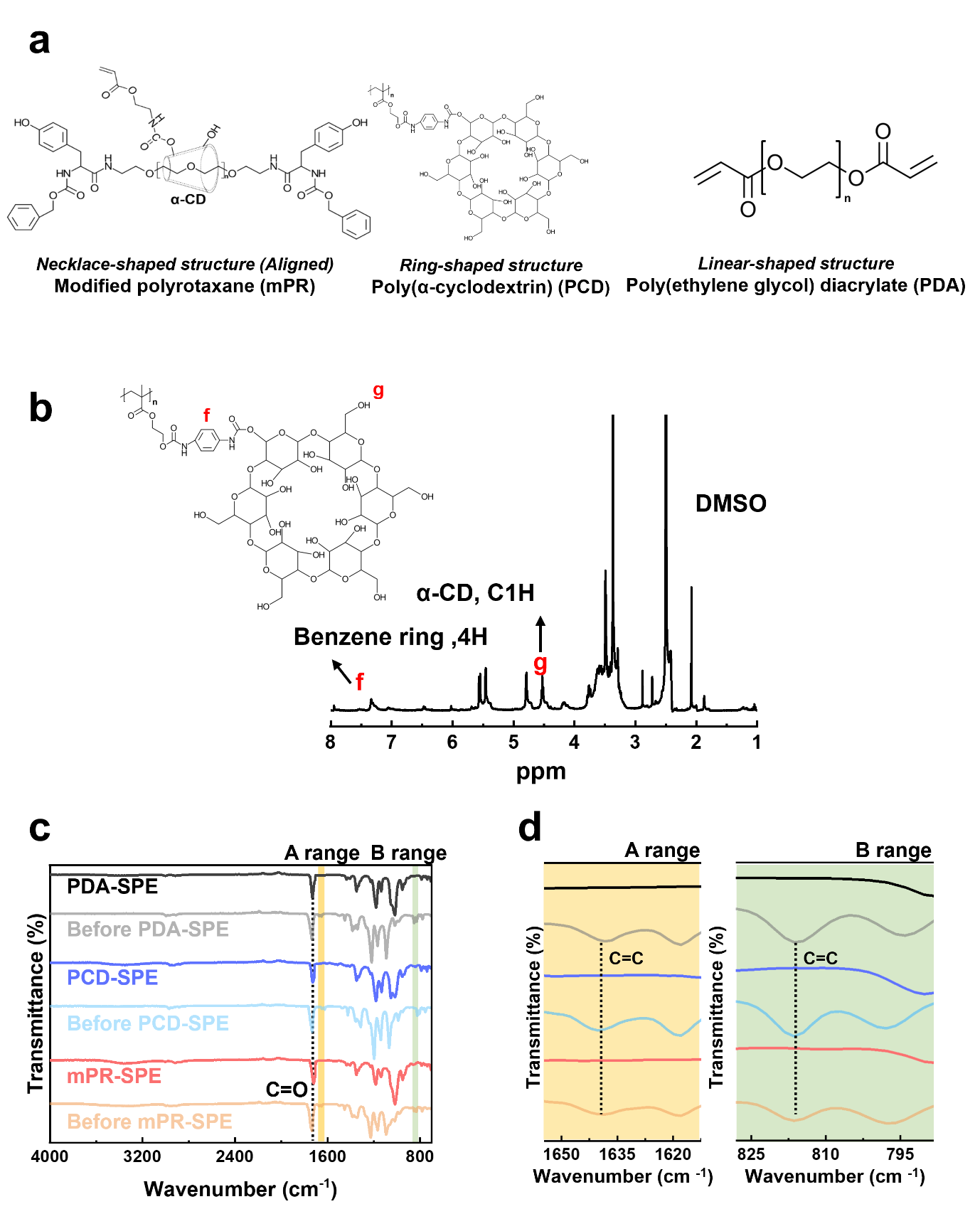


**Fig. S4** (**a**) Chemical structures of the three types of polymers containing acryl group. (**b**) ^1^H NMR spectrum (400 MHz, DMSO-d_6_) of PCD (**c**) FT-IR spectra of mPR-SPE, PCD-SPE and PDA-SPE before and after curing


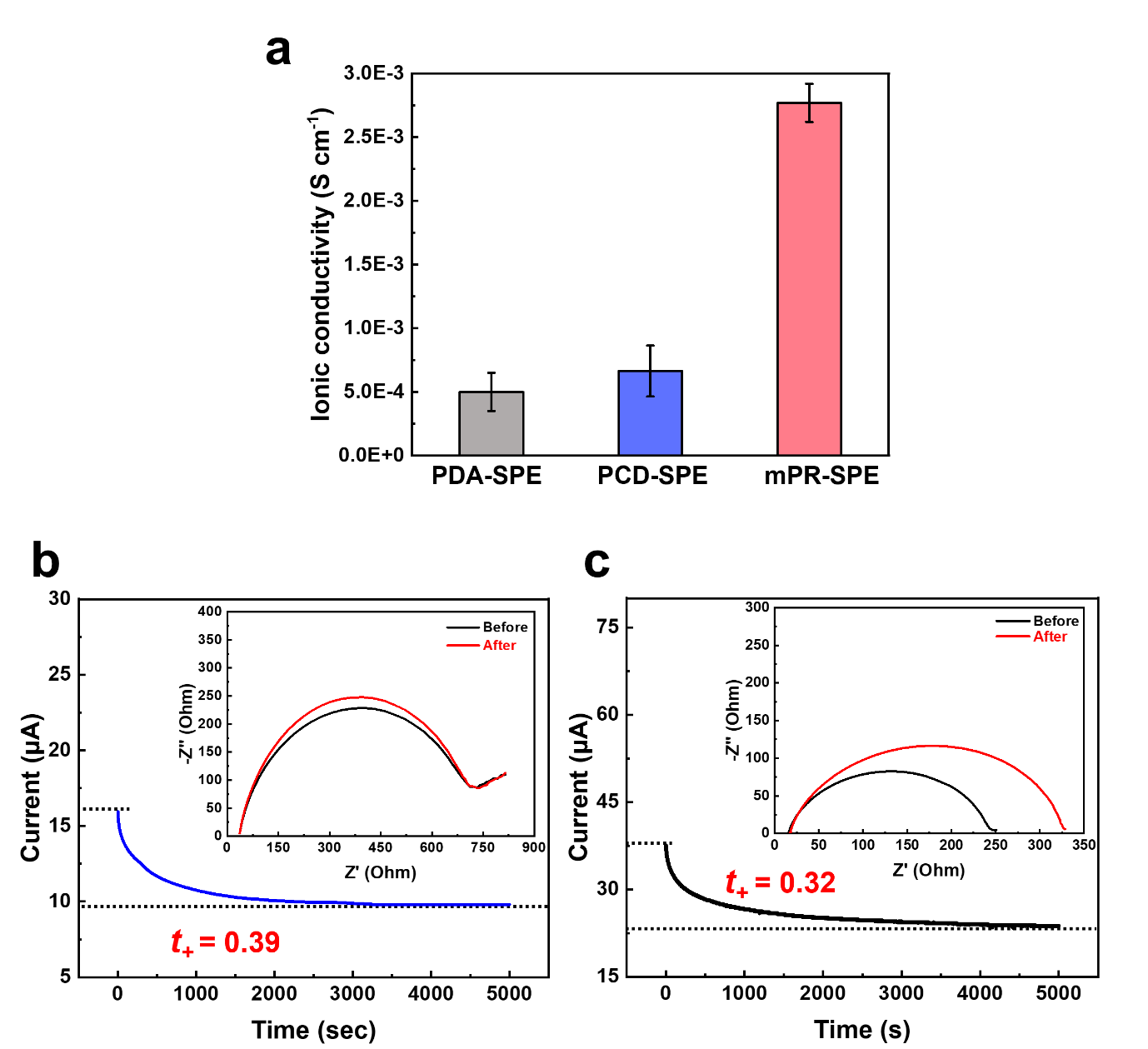


**Fig. S5** (**a**) Ionic conductivity of PDA-SPE, PCD-SPE, and mPR-SPE at 25 °C. (**b**) Li^+^ transference number of PCD-SPE. (**c**) Li^+^ transference number of PDA-SPE


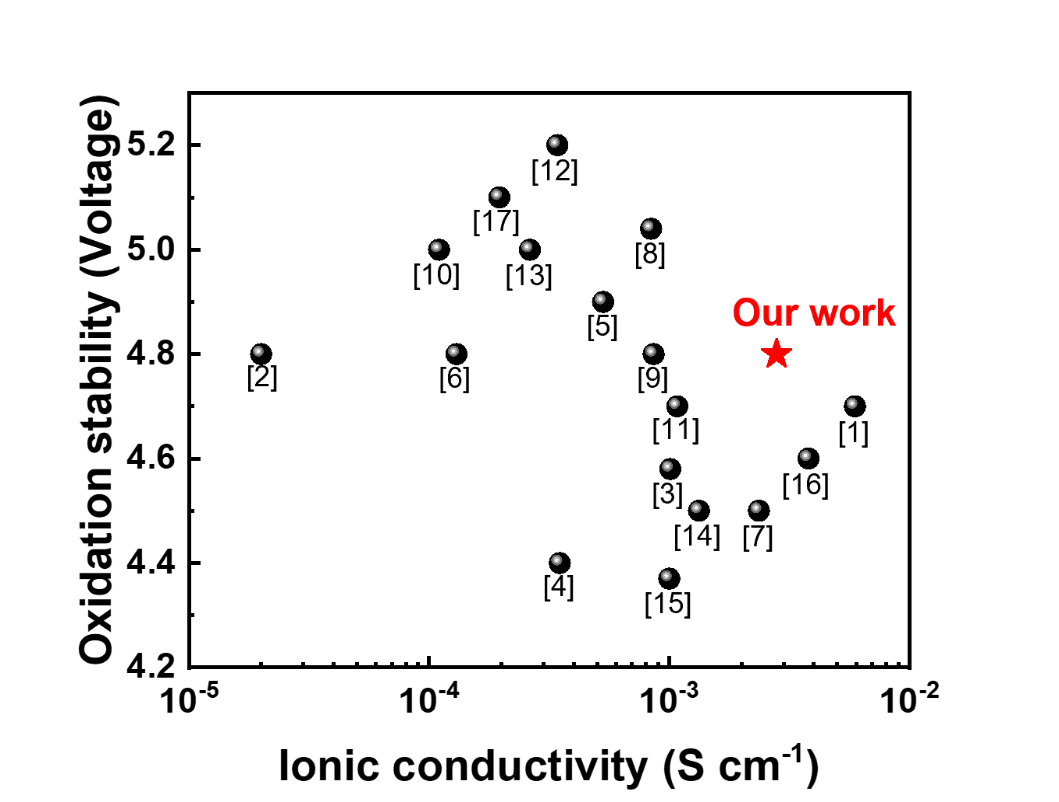


**Fig. S6** Comparison of the ionic conductivity and oxidation stability with those of recently reported polymer-based electrolytes

**Table S1** Comparison of ionic conductivity of recently reproted polymer-based electrolytes

| **Polymer** | **Salt** | **Ionic conductivity at 25 °C (S cm^-1^)** | **Oxidation stability (Voltage)** | **Refs.** |
| --- | --- | --- | --- | --- |
| **mPR** | **LiTFSI** | **2.8E-3**  **(**at 25 °C) | **4.8** | **This study** |
| PRX | LiNO_3_ | 5.93E-3  (at 2 5°C) | 4.7 | [S1] |
| PAN | LiTFSI | 2.0E-5  (at 30 °C) | 4.8 | [S2] |
| HFBA+MBAM | LiTFSI | 1.01E-3  (at 25 °C) | 4.58 | [S3] |
| Elastic epoxy | LiTFSI | 3.5E-4  (at 25 °C) | 4.4 | [S4] |
| P(VDF-HFP) | LiTFSI (EMIMTFSI) | 0.53E-3  (at 20 °C) | 4.9 | [S5] |
| PEGMA | LiDFOB | 1.3E-4  (at 30 °C) | 4.8 | [S6] |
| DOL + PEE | LiTFSI | 2.36E-3  (at 25 °C) | 4.5 | [S7] |
| PALE | LiTFSI | 0.84E-3  (at 25 °C) | 5.04 | [S8] |
| PVCM | LiDFOB | 8.61E-4  (at 25 °C) | 4.8 | [S9] |
| MEDP+EGMEA | LiTFSI | 1.1E-4  (at 25 °C) | 5 | [S10] |
| PEO+PMMA | LiTFSI | 1.08E-3  (at 25 °C) | 4.7 | [S11] |
| PEO+LLZTO | LiTFSI | 1.96E-4  (at 40 °C) | 5.1 | [S12] |
| P(VEC-CEA) | LiTFSI | 2.63E-4  (at 25 °C) | 5.0 | [S13] |
| PVDF-HFP | LiTFSI | 1.33E-3  (at 25 °C) | 4.5 | [S14] |
| PEO+PEGDE+BADGE | LiPF_6_ | 1.0E-3  (at 25 °C) | 4.37 | [S15] |
| PEGMA+UPyMA | LiTFSI | 3.8E-3  (at 25 °C) | 4.6 | [S16] |
| PEGDA+UPyMA | LiTFSI | 3.42E-4  (at 25°C) | 5.2 | [S17] |


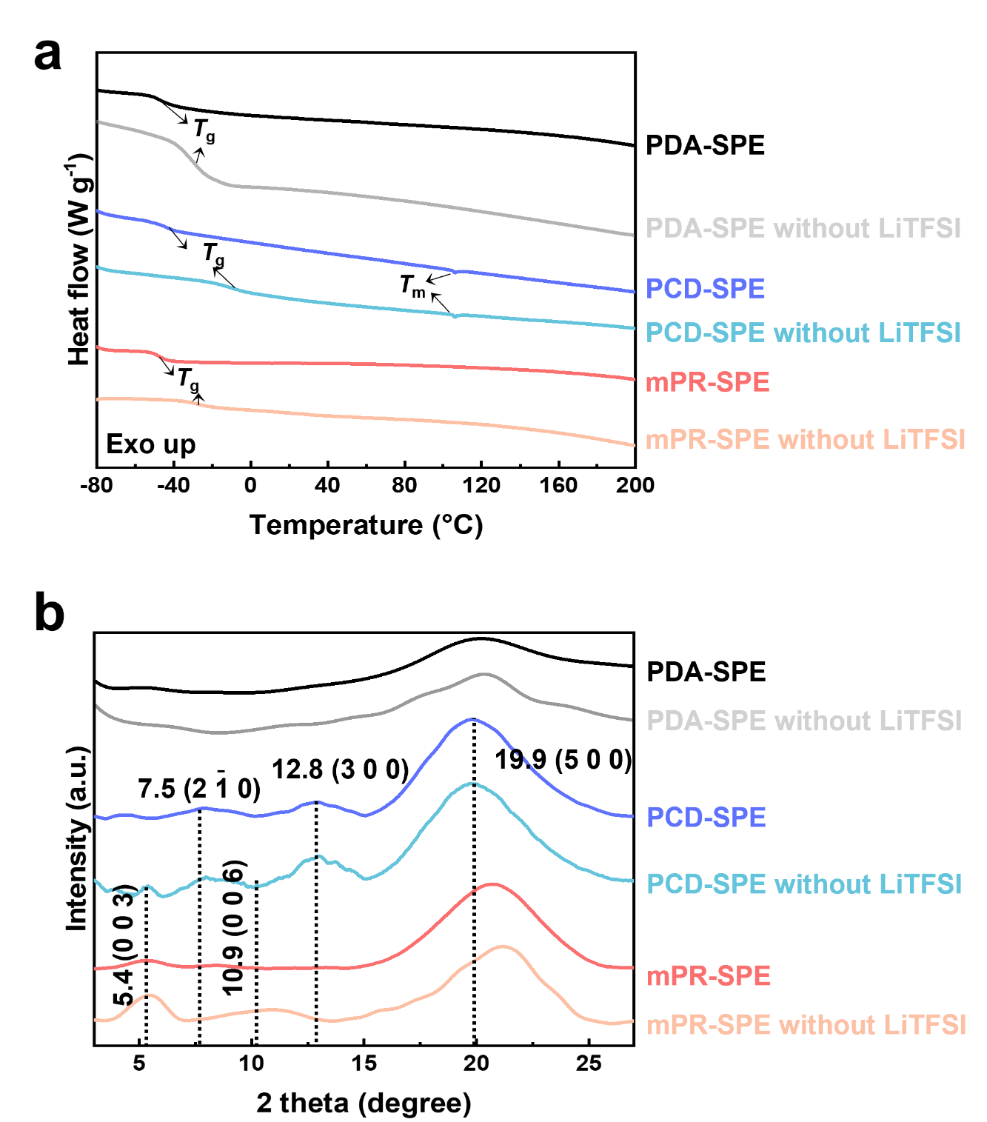


**Fig. S7** (**a**) DSC data of PDA-SPE, PCD-SPE, and mPR-SPE with and without salts. (**b**) XRD data of PDA-SPE, PCD-SPE, and mPR-SPE with and without salts

**Table S2** Transition temperature and thermodynamic variables measured during the 2^nd^ run of the DSC heat flow

| **Sample** | ***T*_g_ (°C)** | ***T*_c_ (°C)** | ***T*_c_ (°C)** |
| --- | --- | --- | --- |
| PDA-SPE | –47 | - | - |
| PDA-SPE  (without LiTFSI) | –31 | - | - |
| PCD-SPE | –44 | - | 106.3 |
| PCD-SPE  (without LiTFSI) | –12 | - | 105.6 |
| mPR-SPE | –45 | - | - |
| mPR-SPE  (without LiTFSI) | –25 | - | - |
| *T*_g_: Glass transition temperature  *T*_c_: Crystallization temperature  *T*_m_: Melting temperature | | | |


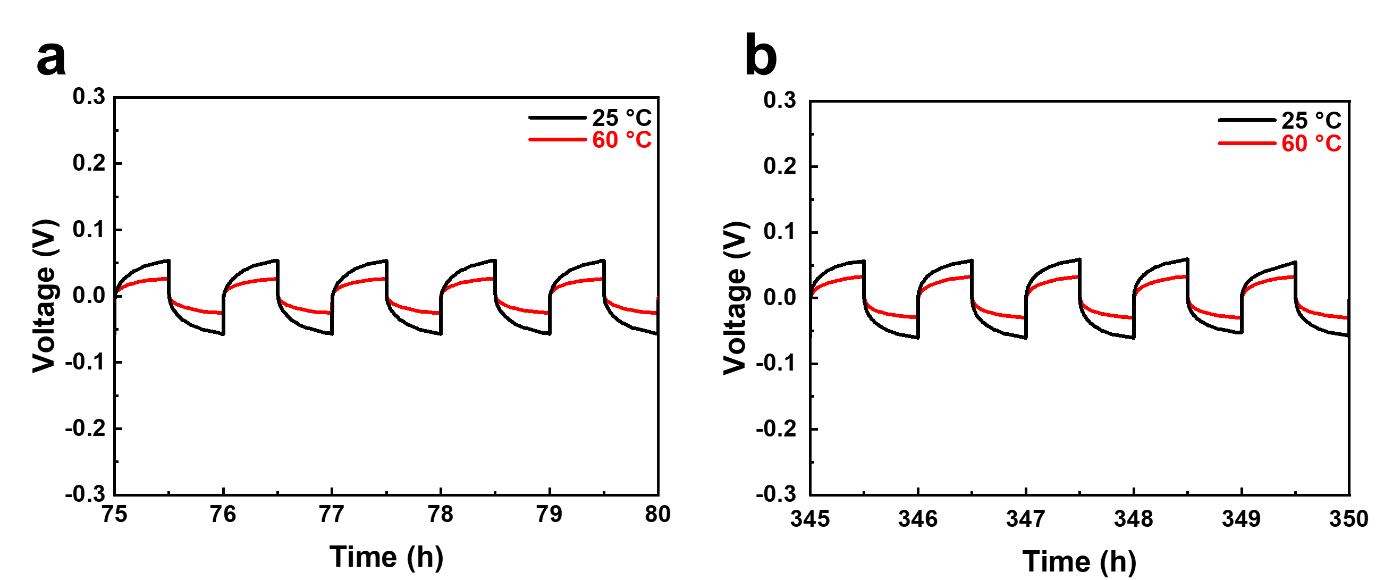


**Fig S8** Enlarged profiles of cycling performance of the symmetric Li cells with mPR-SPE at a current density of 0.2 mA cm^-2^ corresponding to (**a**) 75-80 cycles and (**b**) 345-350 cycles


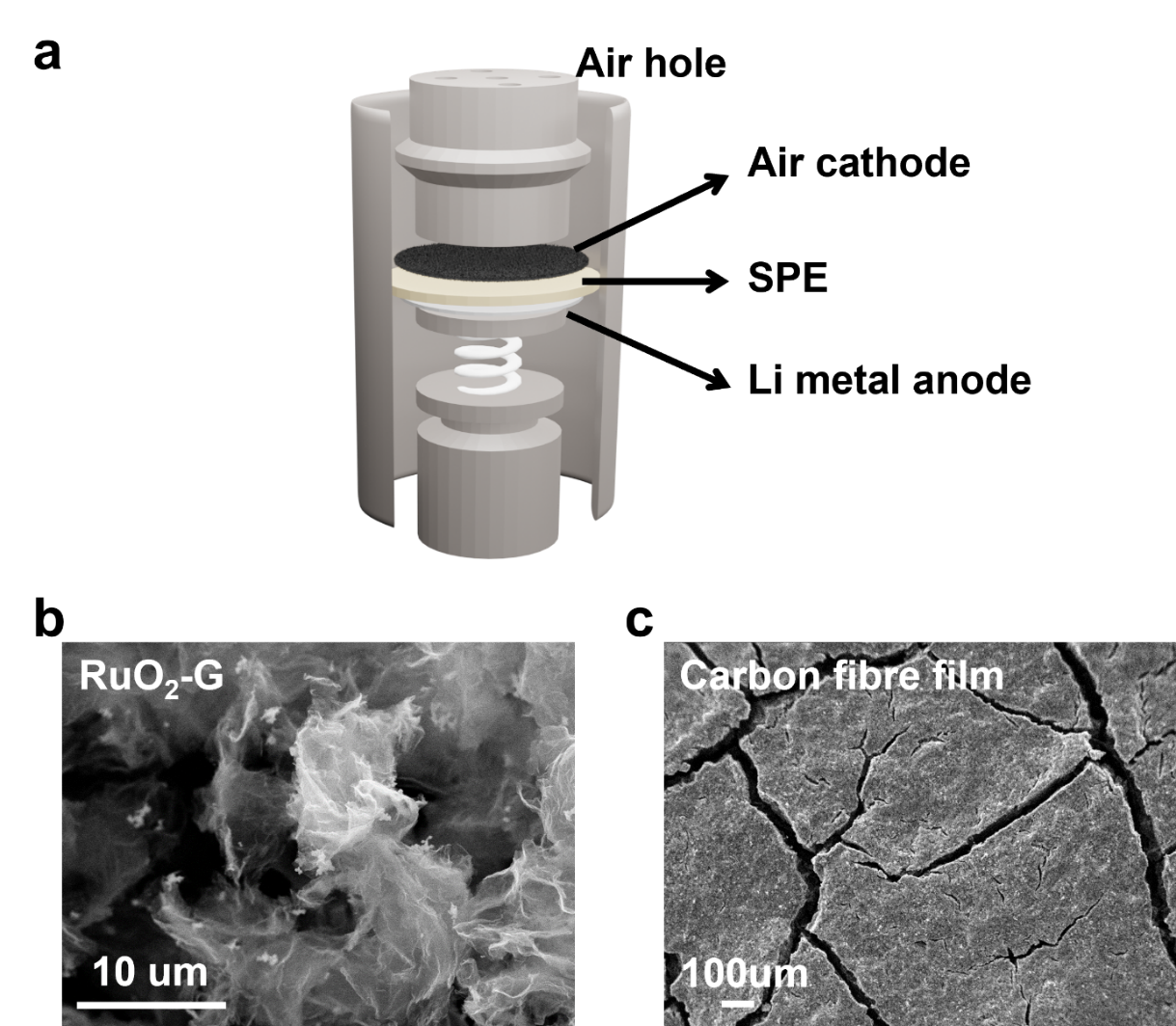


**Fig. S9** (**a**) Main component of solid-state lithium oxygen battery (LOB). SEM images of (**b**) RuO_2_-G catalyst, (**c**) carbon fibre film. Mass of cathode material loading (W_cat_) was calculated by relevant electrode information

cathode surface area = 0.785 cm^2^

loading mass of catalyst = 0.35 mg

Specific capacity (Ah/g_cat_) = current density (A/g_cat_) × 1 h,

W_cat_ = $\frac{0.35 mg}{0.785 cm2}$ = 0.45 mg/cm^2^


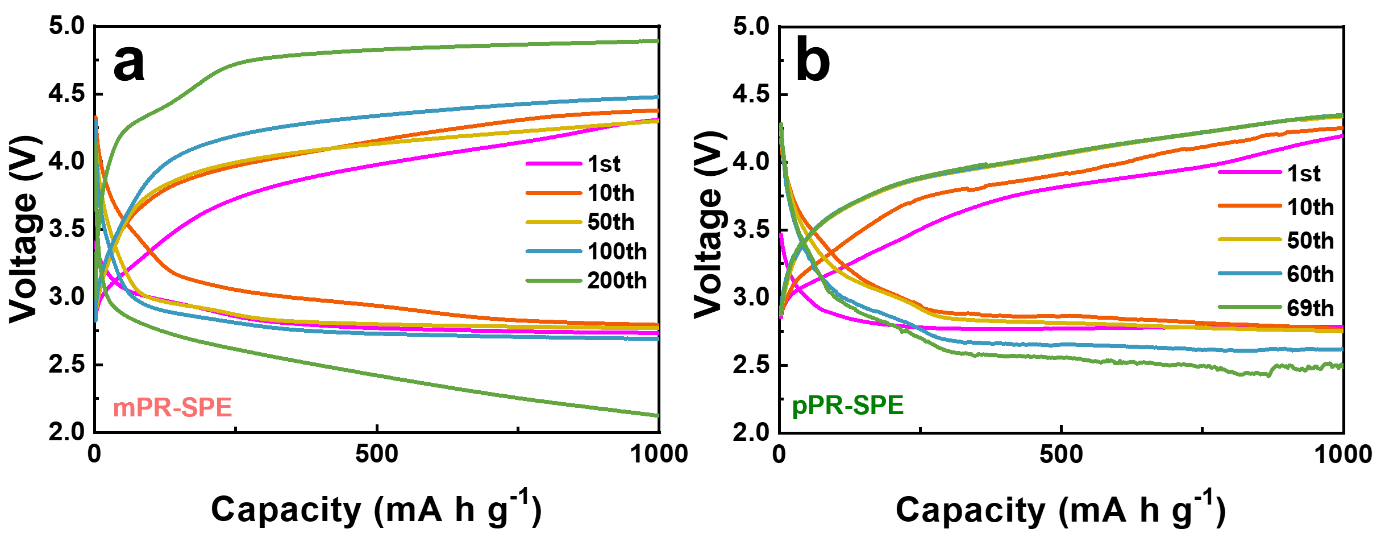


**Fig. S10** Galvanostatic discharge-charge curves of solid-state Li-O_2_ battery cell at a current rate of 500 mA g^−1^ and a fixed capacity limit of 1000 mA h g^−1^ in different polyelectrolytes of (**a**) mPR-SPE and (**b**) pPR-SPE

**Table S3** Comparison of electrocatalytic performances of recently applied polymer-based solid-state LOBs

| Polymer | Catalysts | Limited capacity  (mA h g^–1^) | Current density  (mA g^–1^) | Cycle  (number) | Lithium salt & solvent | Refs. |
| --- | --- | --- | --- | --- | --- | --- |
| **mPR** | **RuO_2_-G**  **Carbon fibre film** | **500** | **100**  **[0.045 mA cm^-2^]** | **300** | **0.75 M LiTFSI**  **in DMSO** | **This study** |
|  |  | **1000** | **500**  **[0.225 mA cm^-2^]** | **202** | **0.75 M LiTFSI**  **in DMSO** | **This study** |
| PVDF-HFP  @SiO_2_-SO_3_Li | Ru@C | 600 | 100 | 370 | 1.0 M LiTFSI  in TEGDME | [S18] |
| PTFE  @XPEG/SNPC | Super P | 1000 | 500 | 277 | 1.0 M LiTFSI  in TEGDME | [S19] |
| MSTP-BQ | CNTs/LiFePO_4_ | 500 | 500 | 200 | LiTFSI  in TEGDME | [S20] |
| SN-PCE  (PVDF-HFP, BHT) | Ru-CNTs | 500 | 200 | 150 | 1.0 M LiCF_3_SO_3_  in DMSO | [S21] |
| PTFE@PS | Super P | 500 | 500 | 149 | 1.0 M LiTFSI  in TEGDME | [S22] |
| P(VDF-HFP)  + silica | Ru-CNTs | 1000 | 500 | 145 | LiTFSI  in DMAc | [S23] |
| PEGMA  +MTA | Pd_3_Co/MWCNT | 500 | 100 | 125 | 1.0 M LiCF_3_SO_3_  in TEGDME | [S24] |
| P(VDF-HFP)  + silica | Ru@Super P | 1000 | 200 | 89 | 1.0 M LiTFSI  in TEGDME | [S25] |
| P(VDF-HFP) | Super P | 500 | 100 | 56 | 1.0 M LiTFSI  in TEGDME | [S26] |
| P(VDF-HFP) | Carbon black | 500 | 500 | 50 | 1.0 M LiTFSI  in TEGDME | [S27] |
| FST-GPE | CNT | 1000 | 500 | 30 | 1.0 M LiTFSI  in TEGDME | [S28] |
| P(VDF-HFP) | SGL Carbon  (gas diffusion layer) | 198 | 200 | 89 | 1.0 M LiTFSI  in TEGDME | [S29] |
| P(VDF-HFP) | Co_3_O_4_@CC | 0.125  (mA h cm^-2^) | 0.025 mA cm^-2^ | 101 | LiTFSI  in DMF, THF | [S30] |
| PEO-based gel polymer (PG) | Carbon paper | 1.0  (mA h cm^-2^) | 0.2 mA cm^-2^ | 110 | 1.0 M LiTFSI  in TEGDME | [S31] |
| PMMA/SiO_2_/PP | CNTs | 0.5  (mA h cm^-2^) | 0.1 mA cm^-2^ | 116 | LiTFSI  in TEGDME | [S32] |
| P(VDF-HFP) | Co_3_O_4_/RuO_2_ | 0.1  (mA h cm^-2^) | 0.1 mA cm^-2^ | 553 | LiClO_4_  in TEGDME | [S33] |


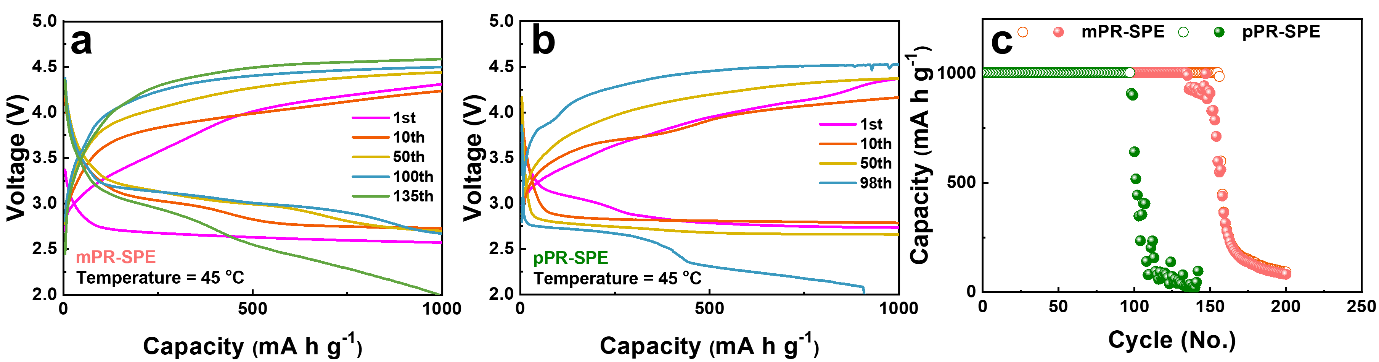


**Fig. S11** Galvanostatic discharge-charge curves of solid-state Li-O_2_ battery cell with (**a**) mPR-SPE and (**b**) pPR-SPE at 45 °C. (**c**) Cycling performance of mPR-SPE and pPR-SPE cell associated with Fig. S11a, b


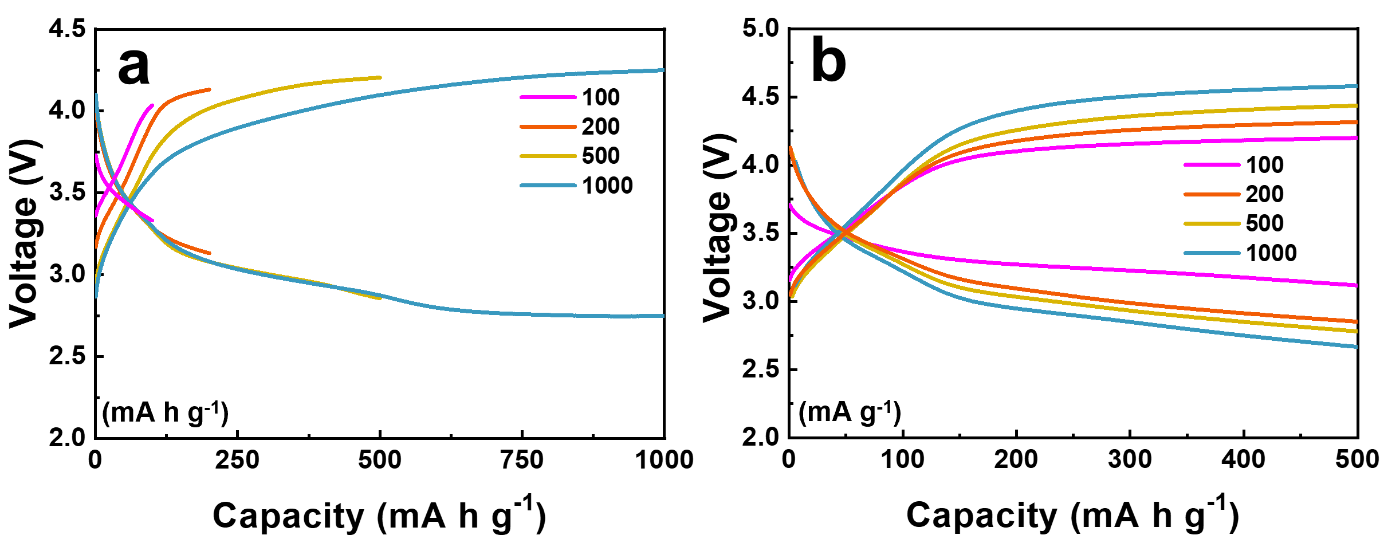


**Fig. S12** (**a**) Galvanostatic cycling of mPR-SPE cell at different capacity limits. (**b**) Galvanostatic cycling of mPR-SPE cell at different current densities at a fixed capacity limit of 500 mAh g^−1^


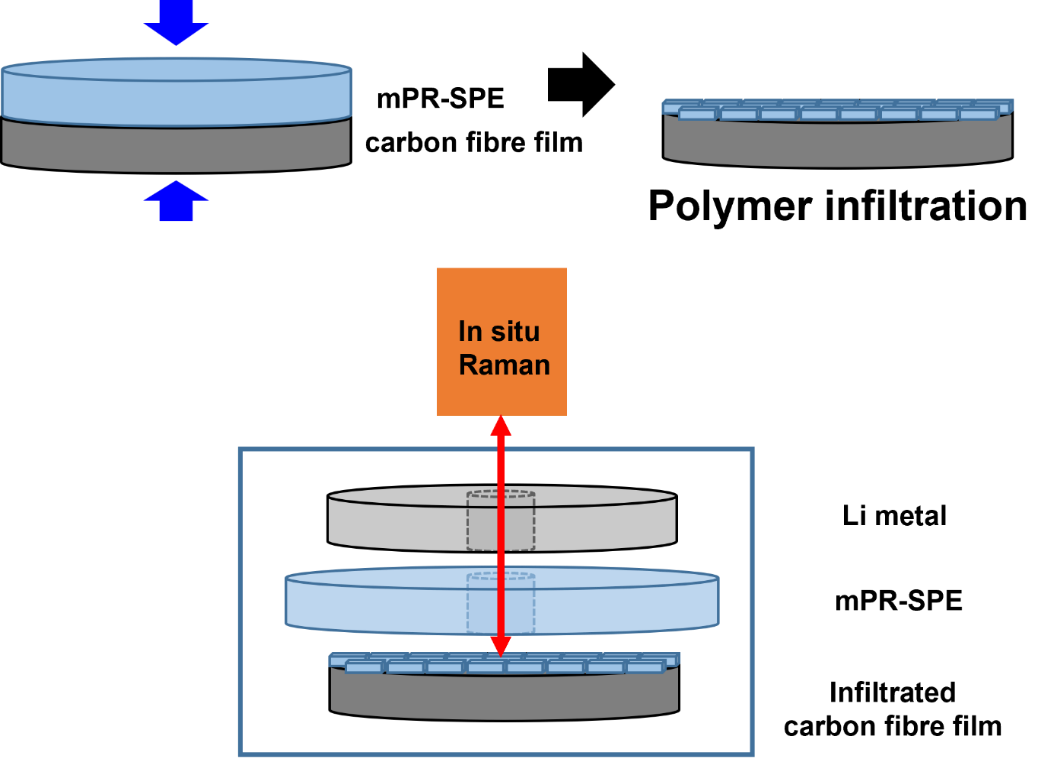


**Fig. S13** Illustration of polymer infiltration and cell structure for in situ Raman analysis


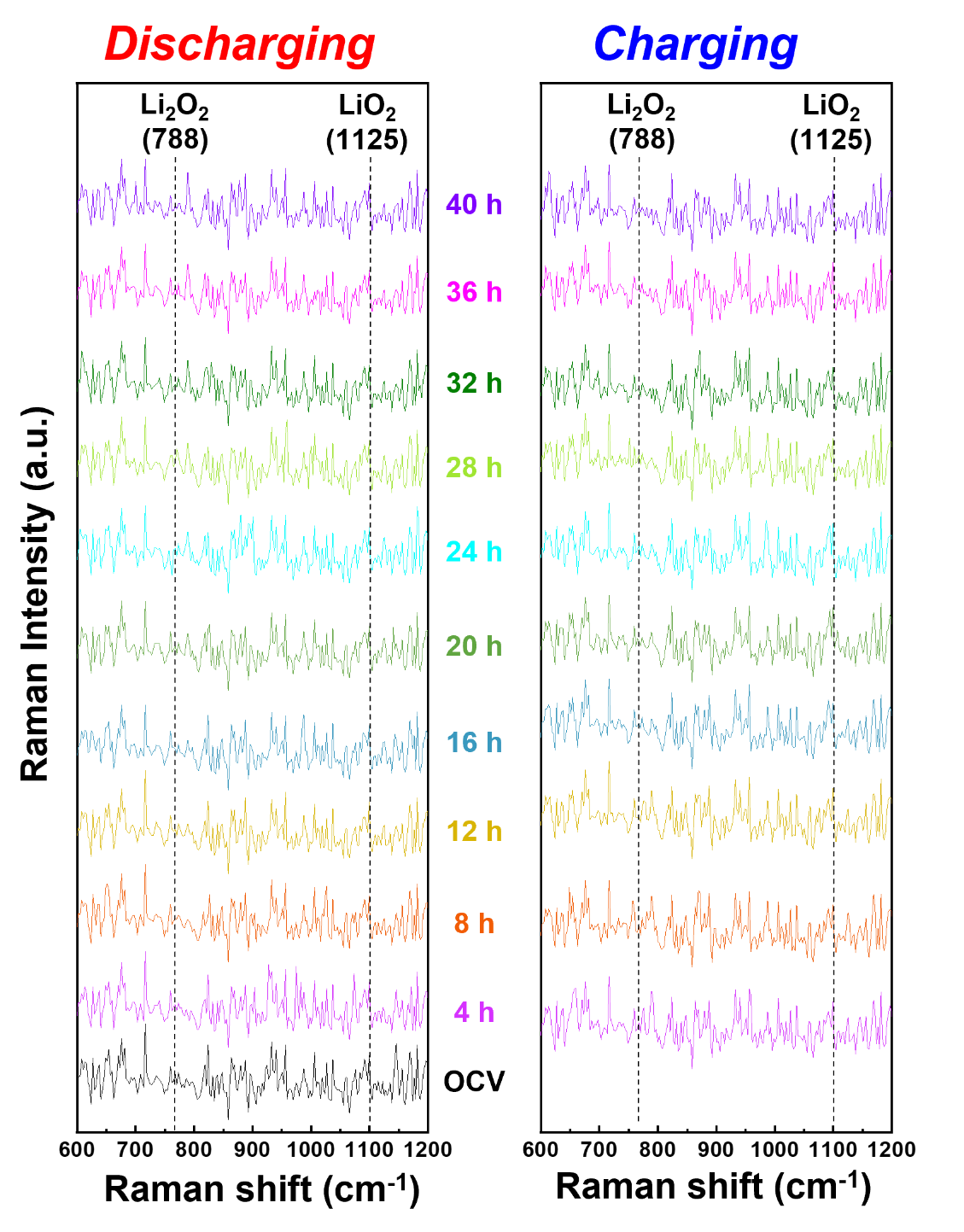


**Fig. S14** In situ Raman spectra from the discharge to the charge process of mPR-SPE cell recorded every 4 h in the galvanostatic discharge–charge curves

**Supplementary References**

1. J. Seo, G.-H. Lee, J. Hur, M.-C. Sung, J.-H. Seo et al., Mechanically interlocked polymer electrolyte with built-in fast molecular shuttles for all-solid-state lithium batteries. Adv. Energy Mater. **11**, 2102583 (2021). <https://doi.org/10.1002/aenm.202102583>
2. C. Liu, R.L. Sacci, R. Sahore, G.M. Veith, N.J. Dudne et al., Polyacrylonitrile-based electrolytes: How processing and residual solvent affect ion transport and stability. J. Power Sources **527**, 231165 (2022). <https://doi.org/10.1016/j.jpowsour.2022.231165>
3. S. Qi, M. Li, Y. Gao, W. Zhang, S. Liu et al., Enabling scalable polymer electrolyte with dual-reinforced stable interface for 4.5 V lithium-metal batteries. Adv Mater. **35**, 2304951 (2023). <https://doi.org/10.1002/adma.202304951>
4. Z. Zeng, X. Chen, M. Sun, Z. Jiang, W. Hu et al., Nanophase-separated, elastic epoxy composite thin film as an electrolyte for stable lithium metal batteries. Nano Lett. **21**, 3611-3618 (2021). <https://doi.org/10.1021/acs.nanolett.1c00583>
5. M. Yao, Q. Ruan, Y. Wang, L. Du, Q. Li et al., A robust dual-polymer@inorganic networks composite polymer electrolyte toward ultra-long-life and high-voltage Li/Li-rich metal battery. Adv. Funct. Mater. **33**, 2213702 (2023). <https://doi.org/10.1002/adfm.202213702>
6. D. Zhang, Y. Liu, Z. Sun, Z. Liu, X. Xu et al., Eutectic-based polymer electrolyte with the enhanced lithium salt dissociation for high-performance lithium metal batteries. Angew. Chem. Int. Ed. **62**, e202310006 (2023). <https://doi.org//10.1002/anie.202310006>
7. J. Zhu, J. Zhang, R. Zhao, Y. Zhao, J. Liu et al., In situ 3d crosslinked gel polymer electrolyte for ultra-long cycling, high-voltage, and high-safety lithium metal batteries. Energy Storage Mater. **57**, 92-101 (2023). <https://doi.org/10.1016/j.ensm.2023.02.012>
8. S. Chai, Z. Chang, Y. Zhong, Q. He, Y. Wang et al., Regulation of interphase layer by flexible quasi-solid block polymer electrolyte to achieve highly stable lithium metal batteries. Adv. Funct. Mater. **33**, 2300425 (2023). <https://doi.org/10.1002/adfm.202300425>
9. M. Bai, X. Tang, M. Zhang, H. Wang, Z. Wang et al., An in-situ polymerization strategy for gel polymer electrolyte Si||Ni-rich lithium-ion batteries. Nat. Commun. **15**, 5375 (2024). <https://doi.org/10.1038/s41467-024-49713-z>
10. S. Wang, Q. Li, H. Gao, H. Cai, C. Liu et al., A polyzwitterion-mediated polymer electrolyte with high oxidative stability for lithium-metal batteries. Small **19**, 2304677 (2023). <https://doi.org/10.1002/smll.202304677>
11. X. Ye, J. Liang, J. Hu, D. Wu, Y. Li et al., An ultra-thin polymer electrolyte for 4.5 V high voltage LiCoO_2_ quasi-solid-state battery. Chem. Eng. J. **455**, 140846 (2023). <https://doi.org/10.1016/j.cej.2022.140846>
12. K. Khan, M.B. Hanif, H. Xin, A. Hussain, H.G. Ali et al., PEO-based solid composite polymer electrolyte for high capacity retention all-solid-state lithium metal battery. Small **20**, 2305772 (2024). <https://doi.org/10.1002/smll.202305772>
13. S. Ma, Y. Zhang, D. Zhang, Y. Zhang, W. Li et al., –C≡N functionalizing polycarbonate-based solid-state polymer electrolyte compatible to high-voltage cathodes. J. Energy Chem. **98,** 422-431 (2024). <https://doi.org/10.1016/j.jechem.2024.06.052>
14. Y. Zeng, L. Zhao, J. Zhang, Q. Li, D. Sun et al., La_2_O_3_ filler's stabilization of residual solvent in polymer electrolyte for advanced solid-state lithium-metal batteries. Small Sci. **3**, 2300017 (2023). <https://doi.org/10.1002/smsc.202300017>
15. S.H. Kim, N. Park, W. Bo Lee, J.H. Park, Functional sulfate additive-derived interfacial layer for enhanced electrochemical stability of PEO-based polymer electrolytes. Small. **20**, 2309160 (2024). <https://doi.org/10.1002/smll.202309160>
16. F. Chen, C. Guo, H. Zhou, M.W. Shahzad, T.X. Liu et al., Supramolecular network structured gel polymer electrolyte with high ionic conductivity for lithium metal batteries. Small **18**, 2106352 (2022). <https://doi.org/10.1002/smll.202106352>
17. H. Wang, J. Song, K. Zhang, Q. Fang, Y. Zuo et al., A strongly complexed solid polymer electrolyte enables a stable solid state high-voltage lithium metal battery. Energy Environ. Sci. **15**, 5149-5158 (2022). <https://doi.org/10.1039/D2EE02904A>
18. H. Wang, J. Song, K. Zhang, Q. Fang, Y. Zuo et al., A strongly complexed solid polymer electrolyte enables a stable solid state high-voltage lithium metal battery. Energy Environ. Sci. **15**(12), 5149-5158 (2022). <https://doi.org/10.1039/D2EE02904AG> Zheng, T. Yan, Y. Hong, X. Zhang, J. Wu et al., A non-newtonian fluid quasi-solid electrolyte designed for long life and high safety Li–O_2_ batteries. Nat. Commun. **14**, 2268 (2023). <https://doi.org/10.1038/s41467-023-37998-5>
19. J. Kim, A. Le Mong, D. Kim, Thin, flexible, and high-performance solid-state polymer electrolyte membranes for Li–O_2_ batteries. ACS Appl. Energy Mater. **6**, 2877-2885 (2023). <https://doi.org/10.1021/acsaem.2c03804>
20. M. Mushtaq, X. Guo, Z. Zhang, Z. Lin, X. Li et al., Dual-function redox mediator enhanced lithium-oxygen battery based on polymer electrolyte. J. Mater. Sci. Technol. **113**, 199-206 (2022). <https://doi.org/10.1016/j.jmst.2021.10.009>
21. J. Wang, G. Huang, K. Chen, X.-B. Zhang, An adjustable-porosity plastic crystal electrolyte enables high-performance all-solid-state lithium-oxygen batteries. Angew. Chem. Int. Ed. **59**, 9382-9387 (2020). <https://doi.org/10.1002/anie.202002309>
22. Z. Li, Y.-E. Liu, S. Weng, X. Wu, X. Yu et al., Oxygen-permeable and moisture-proof membrane for stable Li-O_2_/air batteries in humid working environment. Energy Storage Mater. **58**, 94-100 (2023). <https://doi.org/10.1016/j.ensm.2023.03.018>
23. C.-L. Li, G. Huang, Y. Yu, Q. Xiong, J.-M. Yan et al., Three birds with one stone: An integrated cathode–electrolyte structure for high-performance solid-state lithium–oxygen batteries. Small. **18**, 2107833 (2022). <https://doi.org/10.1002/smll.202107833>
24. S.M. Cho, J. Shim, S.H. Cho, J. Kim, B.D. Son et al., Quasi-solid-state rechargeable Li–O_2_ batteries with high safety and long cycle life at room temperature. ACS Appl. Mater. Interfaces **10**, 15634-15641 (2018). <https://doi.org/10.1021/acsami.8b00529>
25. C. Shu, J. Long, S.-X. Dou, J. Wang, Component-interaction reinforced quasi-solid electrolyte with multifunctionality for flexible Li–O_2_ battery with superior safety under extreme conditions. Small. **15**, 1804701 (2019). <https://doi.org/10.1002/smll.201804701>
26. T. Li, C. Wang, J. Cheng, J. Guo, A. Xiao et al., Janus polymer composite electrolytes improve the cycling performance of lithium–oxygen battery. ACS Appl. Mater. Interfaces **12**, 12857-12866 (2020). <https://doi.org/10.1021/acsami.9b23395>
27. W.-C. Lai, R.-W. Fan, A simple low-cost method to prepare gel electrolytes incorporating graphene oxide with increased ionic conductivity and electrochemical stability. J Electroanal. Chem. **907**, 115889 (2022). <https://doi.org/10.1016/j.jelechem.2021.115889>
28. X. Zou, Q. Lu, Y. Zhong, K. Liao, W. Zhou et al., Flexible, flame-resistant, and dendrite-impermeable gel-polymer electrolyte for Li–O_2_/air batteries workable under hurdle conditions. Small. **14**, 1801798 (2018). <https://doi.org/10.1002/smll.201801798>
29. M. Celik, A. Kızılaslan, M. Can, T. Cetinkaya, H. Akbulut, Electrochemical investigation of PVDF: HFP gel polymer electrolytes for quasi-solid-state Li-O_2_ batteries: Effect of lithium salt type and concentration. Electrochim. Acta **371**, 137824 (2021). <https://doi.org/10.1016/j.electacta.2021.137824>
30. M. Li, Y. Lian, Z. Li, M. Zhu, Q. Qiao et al., An integrated architecture of mutually infiltrated nanoarray cathode and polymer electrolyte improves the performances of solid-state lithium−oxygen batteries. Electrochim. Acta **453,** 142360 (2023). <https://doi.org/10.1016/j.electacta.2023.142360>
31. H.-S. Lim, W.-J. Kwak, S. Chae, S. Wi, L. Li et al., Stable solid electrolyte interphase layer formed by electrochemical pretreatment of gel polymer coating on li metal anode for lithium–oxygen batteries. ACS Energy Lett. **6**, 3321-3331 (2021). <https://doi.org/10.1021/acsenergylett.1c01144>
32. X. Liu, X. Xin, L. Shen, Z. Gu, J. Wu et al., Poly(methyl methacrylate)-based gel polymer electrolyte for high-performance solid state Li–O_2_ battery with enhanced cycling stability. ACS Appl. Energy Mater. **4**, 3975-3982 (2021). <https://doi.org/10.1021/acsaem.1c00344>
33. C. Zhao, J. Liang, Q. Sun, J. Luo, Y. Liu et al., Ultralong-life quasi-solid-state Li-O_2_ batteries enabled by coupling advanced air electrode design with li metal anode protection. Small Methods **3**, 1800437 (2019). <https://doi.org/10.1002/smtd.201800437>
